# Predicting prognosis, immunotherapy and distinguishing cold and hot tumors in clear cell renal cell carcinoma based on anoikis-related lncRNAs

**DOI:** 10.3389/fimmu.2023.1145450

**Published:** 2023-06-09

**Authors:** Chao Hao, Rumeng Li, Zeguang Lu, Kuang He, Jiayun Shen, Tengfei Wang, Tingting Qiu

**Affiliations:** ^1^ Jiangxi Cancer Hospital, The Second Affiliated Hospital of Nanchang Medical College, Jiangxi Clinical Research Center for Cancer, Nanchang, China; ^2^ Department of Radiation and Medical Oncology, Zhongnan Hospital of Wuhan University, Wuhan, China; ^3^ Department of Anesthesiology, Sun Yat-sen University Cancer Center/State Key Laboratory of Oncology in South China, Guangzhou, China; ^4^ Department of Pathology, Dushu Lake Hospital Affiliated of Soochow University, Suzhou, China; ^5^ Afliated Hospital of Integrated Traditional Chinese and Western Medicine, Nanjing University of Chinese Medicine, Nanjing, China

**Keywords:** clear cell renal cell carcinoma, anoikis, immunotherapy, lncRNA, hot and cold tumors, prognostic biomarkers

## Abstract

**Background:**

Clear cell renal cell carcinoma (ccRCC) is the most frequently occurring malignant tumor within the kidney cancer subtype. It has low sensitivity to traditional radiotherapy and chemotherapy, the optimal treatment for localized ccRCC has been surgical resection, but even with complete resection the tumor will be eventually developed into metastatic disease in up to 40% of localized ccRCC. For this reason, it is crucial to find early diagnostic and treatment markers for ccRCC.

**Methods:**

We obtained anoikis-related genes (ANRGs) integrated from Genecards and Harmonizome dataset. The anoikis-related risk model was constructed based on 12 anoikis-related lncRNAs (ARlncRNAs) and verified by principal component analysis (PCA), Receiver operating characteristic (ROC) curves, and T-distributed stochastic neighbor embedding (t-SNE), and the role of the risk score in ccRCC immune cell infiltration, immune checkpoint expression levels, and drug sensitivity was evaluated by various algorithms. Additionally, we divided patients based on ARlncRNAs into cold and hot tumor clusters using the ConsensusClusterPlus (CC) package.

**Results:**

The AUC of risk score was the highest among various factors, including age, gender, and stage, indicating that the model we built to predict survival was more accurate than the other clinical features. There was greater sensitivity to targeted drugs like Axitinib, Pazopanib, and Sunitinib in the high-risk group, as well as immunotherapy drugs. This shows that the risk-scoring model can accurately identify candidates for ccRCC immunotherapy and targeted therapy. Furthermore, our results suggest that cluster 1 is equivalent to hot tumors with enhanced sensitivity to immunotherapy drugs.

**Conclusion:**

Collectively, we developed a risk score model based on 12 prognostic lncRNAs, expected to become a new tool for evaluating the prognosis of patients with ccRCC, providing different immunotherapy strategies by screening for hot and cold tumors.

## Introduction

Renal cell carcinoma is a type of malignant tumor that develops from the epithelial cells lining the renal tubules in the urinary system. Among them, ccRCC is the most common, accounting for about 70% -80% of all RCC, and the peak age of onset is 60-70 years old (1, 2). As ccRCC is not sensitive to chemotherapy and radiotherapy, surgical treatment remains the main treatment for ccRCC (3), but with resection, up to 20% of ccRCC will be eventually developed into recurrence with poor prognosis (4, 5). Despite the widespread use of targeted drugs in the treatment of RCC, the median survival time remains not as good as expected. Therefore, it is of vital necessity to identify markers for early diagnosis and therapeutic targets of ccRCC for individualized treatment.

Anoikis, a special type of programmed cell death, is triggered when normal epithelial cells are deprived of connections to their extracellular matrix (6), which ensures a dynamic balance of normal cell proliferation, differentiation, and apoptosis. Anoikis-resistant tumor cells are prone to facilitate regional or distant metastasis through blood or lymph (7). The transformation and metastasis of malignant tumors are premised on anoikis resistance, which is a characteristic of malignant cells (7–9). Paoli et al. found that when cells adhere to ECM, they can up-regulate the expression of anti-apoptotic proteins such as Bcl-2 and NF-κB through the PI3K/AKT pathway, and down-regulate the expression of pro-apoptotic proteins such as Bad and Bim, thereby inhibiting the occurrence of anoikis (7). Ediriweera et al. also pointed out that the activation of this signaling may be one of the main mechanisms for tumor cells to resist anoikis (10). However, few studies have systematically elucidated the influence of anoikis on ccRCC.

Long non-coding RNA (lncRNA) is closely related to cell function and many diseases (11, 12). At present, studies have demonstrated that lncRNAs are abnormally expressed in a variety of cells and tumor tissues, and can participate in transcription, and gene regulation, affecting tumor cell apoptosis, proliferation, invasion, autophagy and epithelial-mesenchymal transformation (13). Yue et al. reported that lncRNA DLEU1 is involved in the invasion and metastasis of RCC by regulating Akt and EMT pathways. Knockdown of DLEU1 suppressed the progression of RCC (14). It was also investigated that the high expression of lncRNA MALAT1 is significantly related to the malignant degree of glioma and the poor prognosis of patients (15). Besides, Hu et al. demonstrated that lncRNA PLAC2 can target the ribosomal protein L36 of glioma and induce cell cycle arrest (16). However, the critical role of anoikis-related lncRNAs in ccRCC has not been investigated. Therefore, our study may elucidate the mechanism of anoikis and lncRNAs in the prognosis, immune landscape, and drug treatment of ccRCC.

Based on the complexity and role of immunosuppressive tumor microenvironments, researchers have been committed to reversing the “cold” tumors “hot” in recent years to create a more favorable therapeutic environment for tumor immunotherapy and benefit more patients (17). As lncRNA is highly evaluated as a new cancer biomarker at present, we tried to reorganize patients based on ARlncRNAs to effectively identify cold and hot tumors, improve prognosis and increase accurate treatment in clinical practice.

In this study, we first explored the differential expression of ARlncRNAs in ccRCC and their differential expression of potential subtypes in ccRCC. Our model based on 12 ARlncRNAs can effectively and accurately predict and judge the prognosis and drug sensitivity of ccRCC patients. To effectively distinguish between hot and cold tumors of ccRCC, we applied the R package ‘ConsensusClusterPlus’ to divide patients into 2 clusters. Different clusters correspond to different immune microenvironments and respond to different immunotherapy effects.

## Methods

### Data acquisition

The RNA-sequencing transcriptome data and corresponding clinical information are available from the TCGA database (https://portal.gdc.can-cer.gov/). After excluding patients with incomplete clinical information and survival time less than 30 days from further evaluation, 512 ccRCC patients were gathered for this study in the aggregate, and randomly divided into train set and test set according to proportion 1: 1. The characteristics of the 512 ccRCC patients included in this study are shown in (**Supplementary Table 1**). The disease-specific survival (DSS) or progression-free survival (PFS) data of TCGA-ccRCC were downloaded from the UCSC Xena platform (https://xena.ucsc.edu/public/). We obtained 640 anoikis-related genes (ANRGs) integrated from Genecards (https://www.genecards.org/) and Harmonizome (https://maayanlab.cloud/Harmonizome/) dataset (**Supplementary Table 2**).

### Anoikis-related lncRNAs

Based on the 640 ANRGs obtained previously, 233 differentially expressed genes in ccRCC were recognized by the limma R package for subsequent analysis (**Supplementary Table 3**). As a final step, 3973 ARlncRNAs were further evaluated using correlation coefficient > 0.4 and p-value< 0.05 as the thresholds (**Supplementary Table 4**).

### Construction of the ARlncRNA signature model

Univariable Cox regression analysis was investigated to screen out lncRNAs significantly related to the overall survival (OS) of ccRCC patients. 10-fold cross-validation and LASSO Cox regression was applied to reduce the possibility of overfitting. Univariable Cox regression analysis was performed on the ARlncRNAs screened by LASSO, and 12 ARlncRNAs related to prognosis were obtained for modeling. Based on the median value of the risk score, ccRCC patients were categorized into high-risk and low-risk groups. Using the R software package survminer, we calculated the Kaplan-Meier survival curve (https://CRAN.R-project.org/package=survmine). To further evaluate the independent prognostic value of the model, univariable and multivariable analyses of age, gender and grade were further performed. The established model’s ability to make accurate predictions was primarily measured using the C-index.

### Construction and verification of predictive nomogram

Nomogram is a common and powerful tool for evaluating prognosis in oncology and medicine. The nomogram was built based on the R package “rms” (https://CRAN.R-project.org/package=rms). 1-year, 3-year, and 5-year survival calibration curves were drawn to determine whether the predicted and actual results are consistent.

### Tumor microenvironments and immune checkpoint profile analysis

TIMER (18), CIBERSOR (19, 20), QUANTISEQ (21), MCPCOUNTER (21), XCELL (20), and EPIC database (22) were performed to estimate the relative infiltration abundance of immune cells of ccRCC. And the immune cell proportion in tumors was identified by performing CIBERSORT, a gene expression-based deconvolution algorithm to describe the cell constitution of tissues in a more detailed way, then we obtained 22 kinds of immune infiltrating cells, including B cells, neutrophils, eosinophils, etc. Correlation coefficients were calculated for correlation analyses between the immune cell infiltration and risk score. The ESTIMATE algorithm was applied to further investigate the infiltration of tumor cells and stromal cells by utilizing the unique properties of the transcription profiles of cancer samples (23). SSGSEA algorithm in the GSVA package was performed to identify distinct types of immune cells and immune-related functions by transforming marker gene expression patterns into quantities of immune cell populations in ccRCC samples (23, 24). The scores in two groups were visualized in the boxplot generated by the ggpubr package. The expression difference of immune checkpoint genes between the low- and high-risk patient groups was determined by applying “ggpubr” packages of R (https://CRAN.R-project.org/package=ggpubr).

### Drug sensitivity

The Genomics of Drug Sensitivity in Cancer (GDSC) database was applied to explore the drug sensitivity, and the R package “pRRophetic” was used to calculate half-maximal inhibitory concentrations (IC50) for patients with ccRCC by constructing the ridge regression model based on the data from the CCLE for the inserted drugs (25, 26). In order to better individualize therapies in specific tumor subgroups, we performed correlation analysis of risk score and IC50 of four common compounds for ccRCC patients.

### Cluster analysis

To explore the response of patients with ccRCC to immunotherapy, we decided to classify patients into distinct clusters based on CC R package (https://www.bioconductor.org/packages/release/bioc/html/ConsensusClusterPlus.html). PCA, Kaplan-Meier survival analysis, and t-SNE based on the R package were carried out to demonstrate that the classification we constructed can distinguish patients well (27, 28). The immune correlation analysis and drug sensitivity were identified according to the ggplot2 and pRRophetic R packages (26).

### Statistical analysis

Spearman correlation coefficients were calculated for correlation analyses to assess the relationship between drug sensitivity and risk score. All analyses were performed based on R v4.1 software. All p-values are two-tailed, and the threshold to define statistical significance was established as p-value< 0.05 unless otherwise specified.

## Results

### Identification of anoikis-related lncRNAs in patients with ccRCC

**Figure 1:**
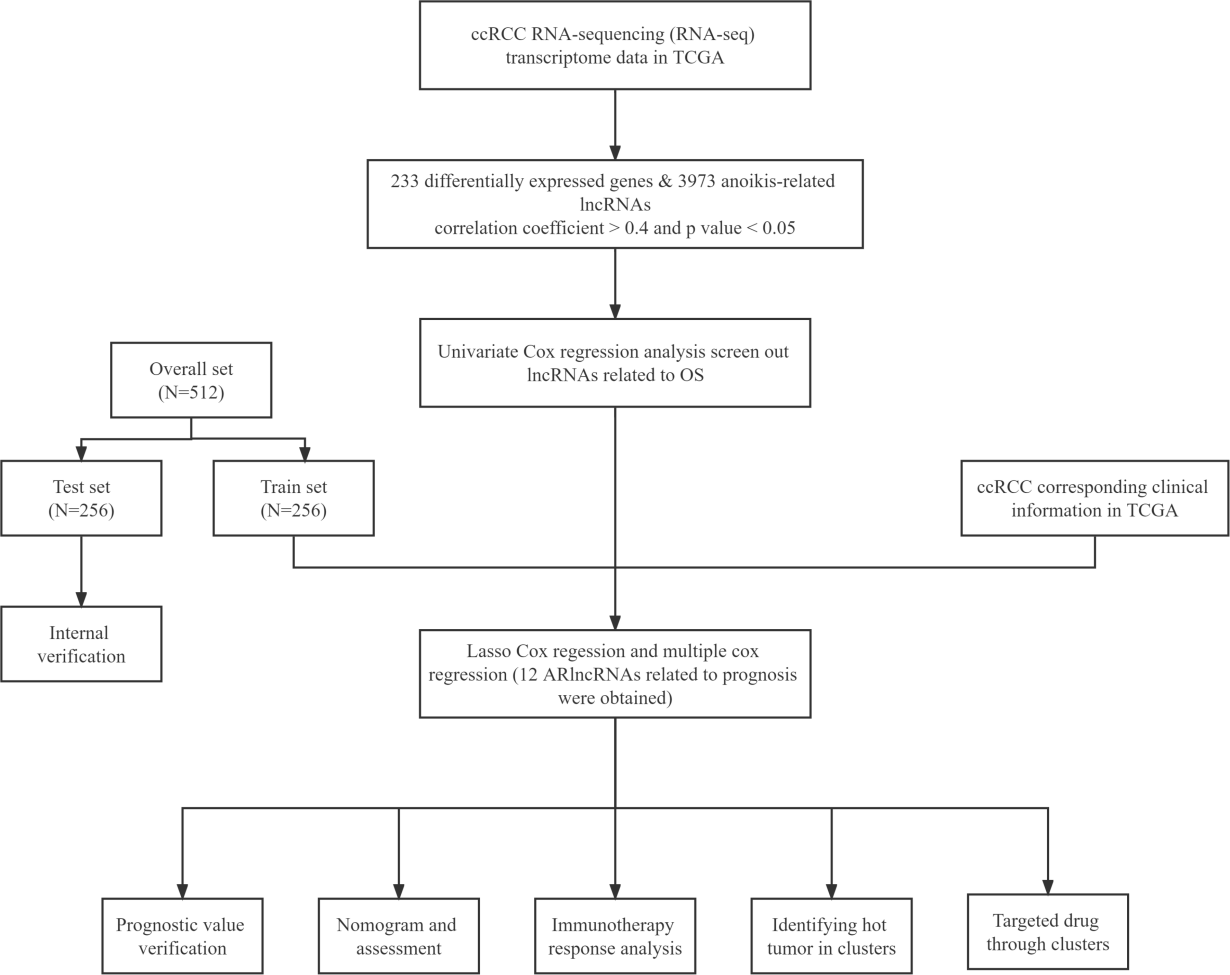
Flow chart of the whole design.

**Figure 2:**
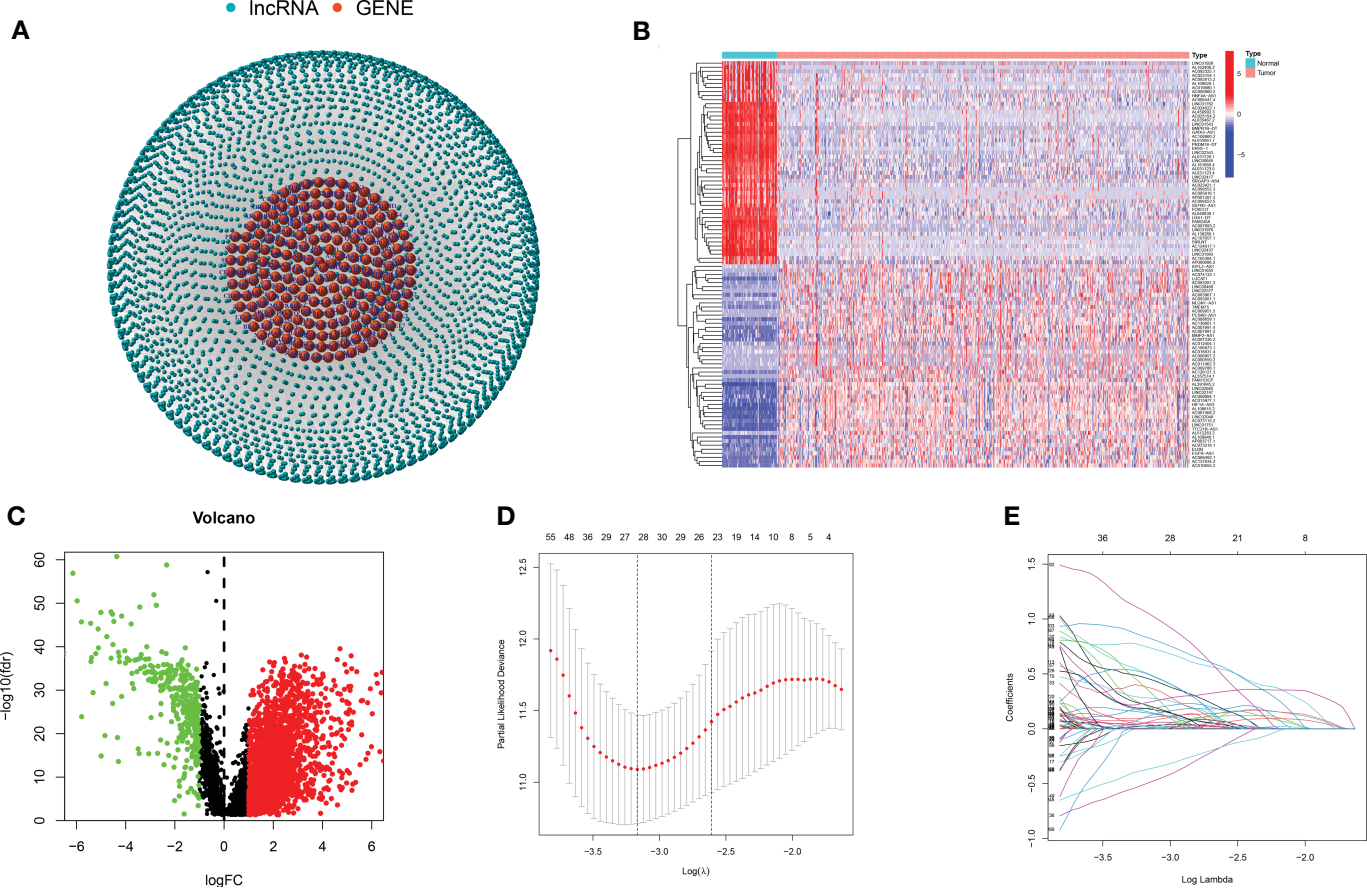
Anoikis-related lncRNAs. **(A)** The co-expression relationship network diagram of anoikis-related genes and lncRNAs. **(B, C)** The heat map and volcano map of differentially expressed lncRNAs. **(D, E)** The LASSO regression was performed, using the minimum criterion.

The flowchart of our study is shown in **Figure 1**. The ARlncRNAs in ccRCC were detected by co-expression analysis, and a co-expression relationship network diagram was constructed (**Figure 2A**). The differentially expressed of lncRNAs were screened out, respectively, with the p< 0.05, |log2FC| > 1 as the screening criteria, and the top 100 differentially expressed lncRNAs with |log2FC| values between normal and tumor tissues were plotted by a heat map (**Figure 2B**). **Figure 2C** demonstrated the differential lncRNA volcano map of ccRCC. As shown in **Figure 2D** and **Figure 2E**, combined with survival data of the ccRCC samples, univariable Cox proportional hazards regression analysis and the LASSO Cox regression analysis with 10 times cross and verification were applied. Then multi-factor cox regression analysis was performed to obtain the lncRNAs used for modeling and the corresponding coefs in **Supplementary Table 5**.

### Establishment of anoikis-related lncRNA model

Based on regression coefficients obtained by the multivariable Cox regression analysis, 12 lncRNAs were input to establish the following risk score formula:


$$\begin{array}{l} \;Risk\;Score\;=\;LINC02609\times \;\left(1.15935260279794\right)\;+\;AC007637.1\\ \times \;\left(-\;1.13652029010858\right)\;+\;ELDR\;\times \;2.00073479059889\;)\\ +\;AC107021.2\;\times \;\left(\;0.261485217934968\;\right)\;+\;AL022238.2\\ \times \;\left(\;0.691751913458257\;\right)\;+\;AC005899.7\;\\ \times \;\left(\;0.916443655715418\;\right)\;+\;LINC01522\\ \times \;\left(\;1.51606402310246\;\right)\;+\;MYOSLID\\ \times \;\left(\;0.759701583473995\;\right)\;+\;AC002070.1\\ \times \;\left(\;-\;0.691247797327996\;\right)\;+\;AC135178.2\\ \times \;\left(\;1.3815487184505\;\right)\;+\;AL590822.3\\ \times \;\left(\;0.93143791519371995\;\right)+AL355922.1\\ \times \left(0.953715193317185\right) \end{array}$$

**Figure 3:**
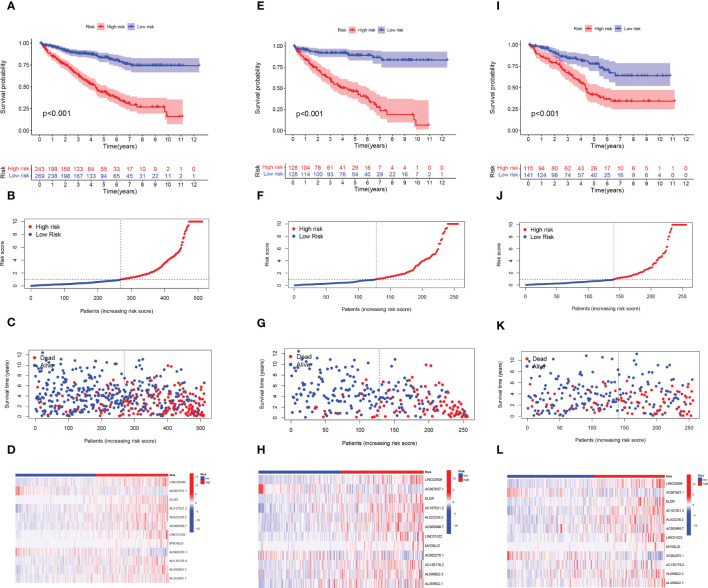
Construction of the ARlncRNA signature model in the train, test, and overall test. Overall survival analysis, risk score distribution, individual survival status, and heat map of 12 ARlncRNAs expression in high and low-risk groups for **(A-D)** the overall set, **(E-H)** the train set, and **(I-L)** the test set.

Patients with ccRCC were randomly assigned to the test and train set and further separated into high- and low- groups based on the median risk score. **Figures 3A, E, I** revealed that OS was significantly higher in the low-risk group compared with the high-risk subgroup in the overall, train, and test sets. The distribution of risk scores and survival status showed that higher risk scores indicated more deaths in these groups of patients with ccRCC (**Figures 3B, C, F, G, J, K**). The heat map results showed that except for AC007637.1 and AC002070.1, the other 10 lncRNAs were well expressed in the high-risk group (**Figures 3D, H, L**). Next, we analyzed the progression-free survival (PFS) and disease-specific survival (DSS) for both high- and low-risk groups (**Supplementary Figure 1**).

### Model validation

**Figure 4:**
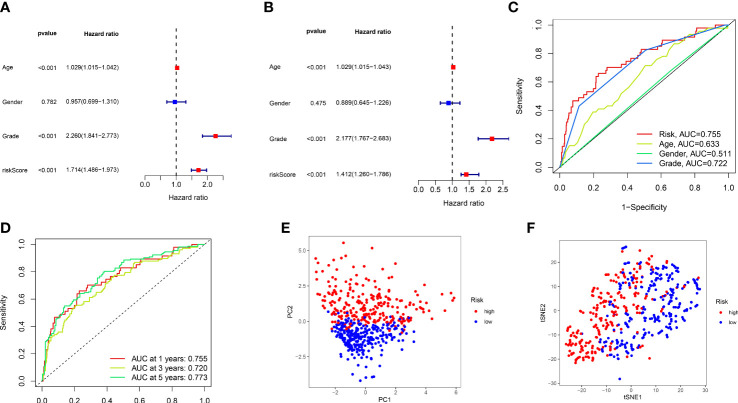
The validation of 12 lncRNAs prognostic model. **(A, B)** Single-factor and multi-factor Cox regression analysis of the risk score combined with clinical features. **(C)** Comparison of ROC curves for risk score and other clinical factors. **(D)** The ROC curves for 1-, 3-, and 5-year overall survival predictions by the risk score model in the training set. **(E, F)**, PCA, and t-SNE analyses of the train set.

On behalf of exploring the predictive accuracy of the model for prognosis, single-factor and multi-factor Cox regression analyses were taken to indicate that the risk score is an independent factor in predicting the prognosis of ccRCC patients (P< 0.001, **Figures 4A, B**). Next, the ROC curves of the constructed risk score were plotted combined with other clinical features, and it was proven that especially risk score had the highest AUC (0.755) among these factors, indicating that our constructed model had higher accuracy for the prediction than other clinical features (**Figure 4C**). It was revealed by the **Figure 4D** that 1-year, 3-year, and 5-year survival with the AUC values of 0.755, 0.720, and 0.773, respectively, performing a strong ability to predict. PCA and t-SNE analyses were applied to verify that the risk score can significantly distinguish patients (**Figures 4E, F**), demonstrating that the risk model based on the expression profile of 12 ARlncRNAs was a potential prognostic marker. The C-index suggests that the risk score outperformed other factors in predicting the outcome (**Supplementary Figure 2**).

### The model guides the clinical treatment of different types of patients

**Figure 5:**
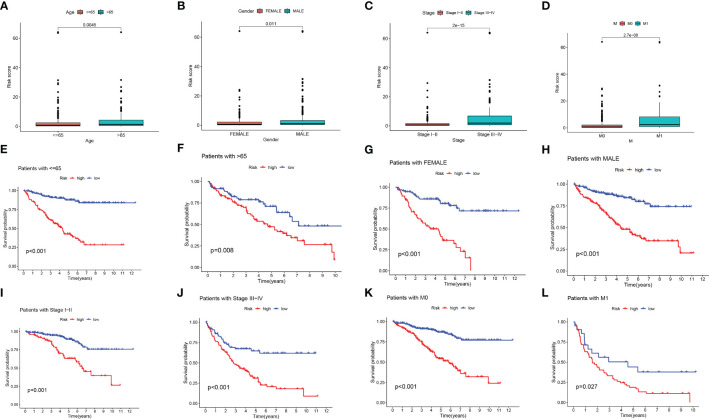
Risk score difference analysis of different clinical features. **(A-D)** Box line diagram and **(E-L)** Kaplan-Meier survival curves in groups stratified by gender, age, stage, and M status.

We performed a score difference analysis of different ages, stages, genders, and hematogenous metastasis. **Figures 5A–D** revealed a higher risk score degree in ccRCC patients older than 65 years old, male, STAGE III-IV, and m1. The KM survival analysis demonstrated that patients in the low-risk group, stratified by age, gender, stage, and presence of metastasis (**Figures 5E–L**), had longer overall survival, indicating that our model was applicable to patients with various clinical characteristics of ccRCC.

### Construction and verification of predictive nomogram

**Figure 6:**
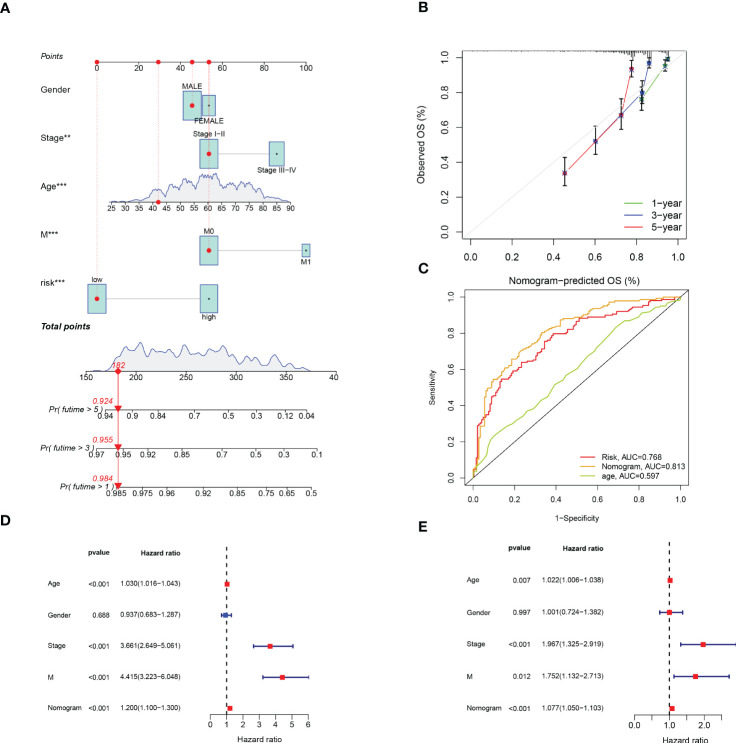
Construction and Verification of Predictive Nomogram. **(A)** The nomogram for predicting 1-year, 3-year, and 5-year survival rates (‘***’ p< 0.001). **(B)** The calibration curves of the nomogram. **(C)** ROC curve of risk score and clinical features. **(D, E)** The univariable and multivariable Cox analysis of nomogram. **p < 0.01; ***p < 0.001.

In addition, we developed a prognostic nomogram that can estimate the probability of patient survival. For instance, with a score of 182, the predicted survival rates were 0.984, 0.955, and 0.924 for 1, 3, and 5 years, respectively (**Figure 6A**). The calibration curves evaluated the good agreement with the ideal model (gray curve) and nomogram prediction (**Figure 6B**). ROC curve showed that nomogram had the strongest predictive value (**Figure 6C**). Combined with clinical features, univariable and multivariable Cox analysis were performed to demonstrate that Nomogram also had independent prognostic survival ability (**Figures 6D, E**).

### Risk score and Immune infiltration landscape evaluation

**Figure 7:**
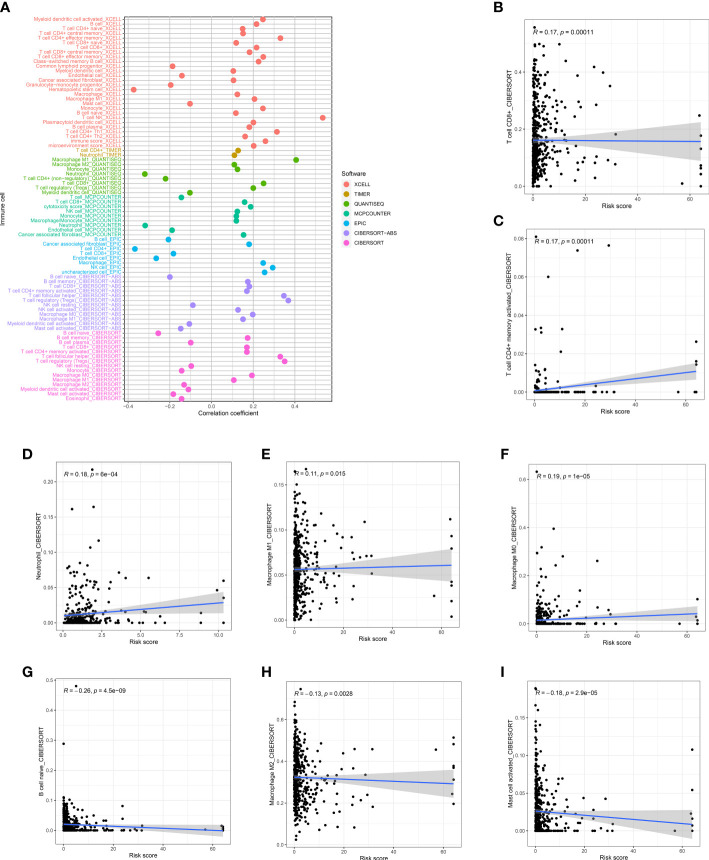
Risk score and Immune infiltration landscape evaluation. **(A)** Evaluation of immune cell infiltration in two groups. The correlation between the risk score and infiltration of CD8+T cells **(B)**, CD4+T cells **(C)**, neutrophils **(D)**, M1 macrophages **(E)**, M0 macrophages **(F)**, naïve B cells **(G)**, M1 macrophages **(H)**, and activated mast cells **(I)** was examined.

The correlation of risk score and TME cell infiltration was investigated by applying several algorithms (**Figure 7A**), showing that the relationship between immune cells and high-risk groups on different platforms is closer. We further analyzed the correlation between immune cells and risk scores in the CIBERSORT platform. From the performance in **Figures 7B-I**, T cell CD8 +, CD4 + memory activated, Macrophage M0, Macrophage M1, and Neutrophil were positively correlated with the risk score, while B cell naïve, Macrophage M2 and Mast cell activated were negatively correlated with the risk score.

**Figure 8:**
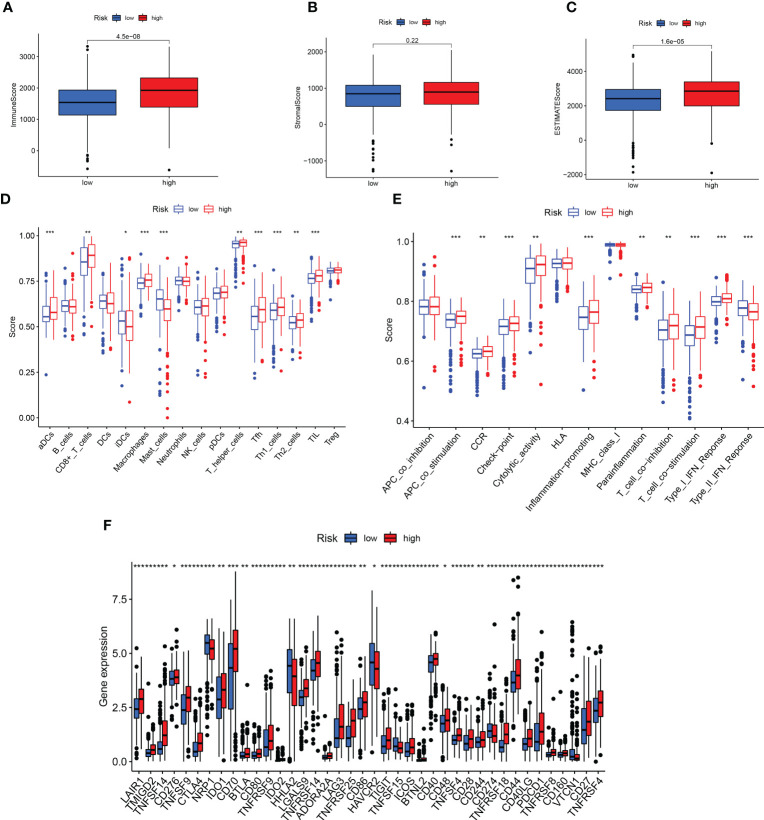
Prediction of immunotherapy. **(A-C)** Differences in TME scores between high- and low-risk groups. **(D, E)** Analysis of immune cells and related functions in two groups. **(F)** Differential expression of immune checkpoint in two groups. **p < 0.01; ***p < 0.001.

The tumor and its environment are mutually interdependent and antagonistic to one another. According to the TME score, the low-risk group had a higher immune score and estimated score, nevertheless, the stromal score did not significantly differ between the two groups (**Figures 8A-C**). Based on the infiltration abundance of the different immune cell populations in the ccRCC TME quantified by ssGSEA analysis, the proportion of immune cell subsets, such as CD8 + T cells, T helper cells, and TIL, increased significantly in the high-risk group (**Figure 8D**). Several immune pathways such as Type I IFN Response, T cell co-stimulation, and T cell co-inhibition had significant differences in the high and low-risk groups (**Figure 8E**). We further analyzed the differences in immune checkpoint expression between the high- and low- risk groups and found that only *HAVCR2* and *CD160* were highly expressed in the low-risk group, and the rest were highly expressed in the high-risk group (**Figure 8F**).

### Exploration of an individualized clinical treatment plan for two groups

**Figure 9:**
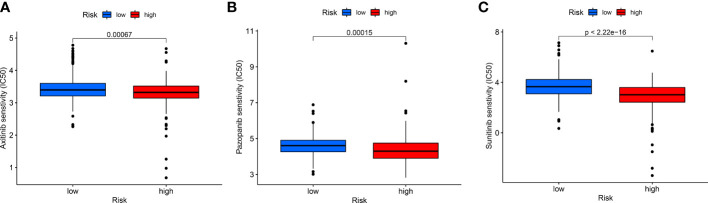
The differences in drug sensitivity of patients. **(A)** Axitinib, **(B)** Pazopanib, and **(C)** Sunitinib.

The sensitivity analysis of three common targeted drugs displayed that the IC50 of Axitinib, Pazopanib, and Sunitinib in the high-risk group was lower (**Figures 9A-C**), indicating that the targeted drugs may have a better therapeutic effect on high-risk groups. There was valuable guiding significance for the medication of patients with ccRCC in actual clinical practice.

### Unsupervised cluster analysis distinguished hot and cold tumors

**Figure 10:**
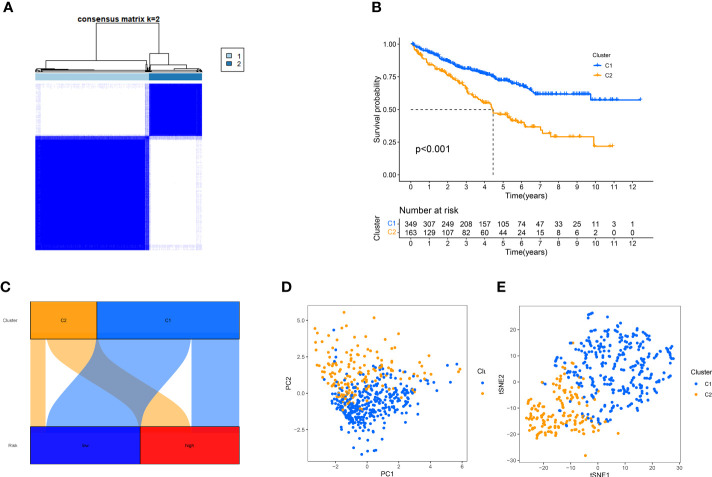
Using consensus clustering analysis, patients can be grouped into two clusters. **(A)** Consensus Cluster Plus divides patients into two categories. **(B)**, Kaplan-Meier survival analysis. **(C)** Correspondence between typing and high and low risk groups. **(D, E)** PCA and t-SNE analyses were performed for the two clusters.

Despite the important value of the risk score we established for tumor prognosis and systemic treatment, it cannot effectively identify hot and cold tumors. Several studies have proven that cluster analysis can be applied to distinguish hot and cold tumors and guide immunotherapy (29–32). Based on the expression levels of Anoikis-Related lncRNAs, ccRCC patients were classified into two clusters using consensus clustering analysis (**Figure 10A**, **Supplementary Figure 2**). Survival curve analysis demonstrated that cluster 1 was associated with a favorable prognosis (**Figure 10B**). Moreover, Cluster1 was associated with a low-risk score, while cluster 2 was associated with high-risk score (**Figure 10C**), indicating that the cluster may be strongly related to the risk score. PCA and t-SNE analysis were carried out to demonstrate that there were significant differences in the two clusters (**Figures 10D, E**).

**Figure 11:**
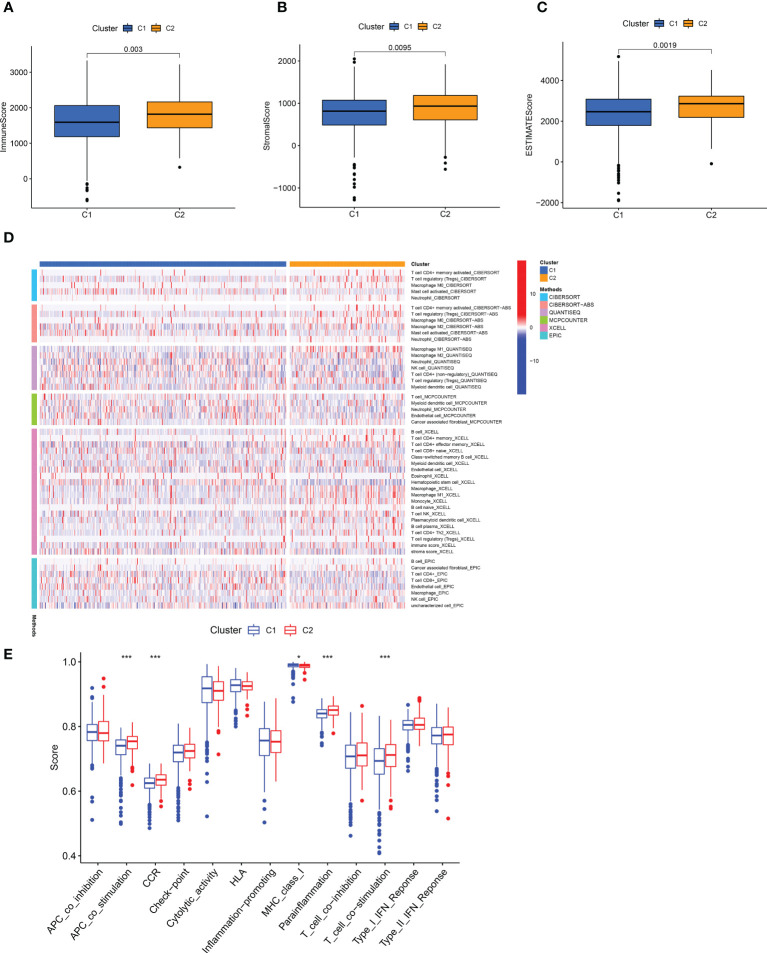
Infiltration of different immune cells in two clusters. **(A-C)** The degree of the immune score, stromal score, and estimated score in distinct clusters. **(D)** A Heat map of immune cells in two clusters based on different platform algorithms. **(E)** The immune-related functions of two clusters were analyzed by ssGSEA analysis. *p < 0.05; **p < 0.01; ***p < 0.001.

To better illustrate the role of a cluster in TME landscapes, we also inspected the implication between cluster and immune infiltration. Cluster 2 showed a high degree of an immune score, stromal score, and estimated score (**Figures 11A-C**). Then, we compared the ssGSEA score of immune cells and pathways using the Wilcoxon test and found that immune cells such as Myeloid dendritic cell and CD8 + T cells were significantly higher in the cluster 1 (**Figure 11D**), and the immune functions such MHC class I was significantly related to the cluster 1 (**Figure 11E**). These analyses suggested that cluster 1 may be summarized as the hot tumor, possibly featured by the good response to immunotherapy, leading to individual immunotherapy regimens in ccRCC.

### Therapeutic drug treatments guided by distinct clusters

**Figure 12:**
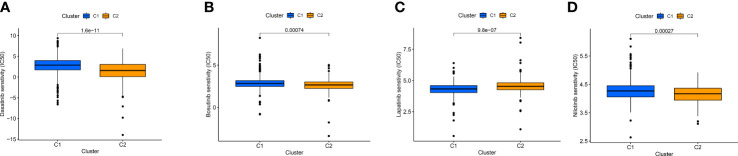
IC50 of Dasatinib **(A)**, Bosutinib **(B)**, Nilotinib **(C)**, and Lapatinib **(D)** targeted drugs in distinct clusters.

By comparing drug sensitivity, we found that Dasatinib, Bosutinib, and Nilotinib were more sensitive in cluster 2, and Lapatinib was more sensitive in cluster 1, which can further help guide the use of clinical targeted drugs (**Figures 12A-D**).

## Discussion

In this study, a risk-scoring model was developed using 12 prognostic lncRNAs. The model’s accuracy was assessed through ROC curve analysis, as well as univariable and multivariable analyses. In addition, we found that the survival rate was lower for individuals in the high-risk group than the low-risk group. However, people in the high-risk group had a lower IC50 of targeted drugs, meaning they were more sensitive to drugs. In addition, previous research has demonstrated that immune cell infiltration was associated with immune efficacy (33, 34). Our findings demonstrated that there are significant variations in immune cell infiltration observed between the high and low-risk groups. It shows that the model we established can provide some thinking and help for immunotherapy of patients with ccRCC.

We established a novel signature based on 12 prognostic ARlncRNAs. Among the 12 ARlncRNAs in the signature, LINC02609, ELDR, AC107021.2, AL022238.2, AC005899.7, LINC01522, MYOSLID, AC135178.2, AL590822.3 and AL355922.1 were highly expressed in the high-risk group. The high expression of LINC02609 not only easy to appear in advanced and graded tumor tissues with distant metastasis but also had significant prognostic potential (35). The human ELDR gene highly expressed in neuronal stem cells (36). Subhayan et al. proved the important role of lncRNAs ELDR in cancer, interacted with RNA binding protein ILF3, and enhanced ILF3-Cyclin E1 signaling to enhance cancer growth (37). Previous studies have shown that MYOSLID was a new serum response factor-dependent lncRNA that regulated VSMC proliferation, apoptosis and differentiation (38, 39). Zhao et al. suggested that MYOSLID played a vital role in vascular remodeling, the basis of the pathogenesis of vascular diseases (38). Ac007637.1 and Ac107021.2 had been proven to be prognostic factors of cancer in several studies, and the risk model respectively based on two lncRNAs that could reliably forecast the prognosis of patients had been successfully constructed (40, 41). The mechanism of AL022238.2, AC005899.7, LINC01522, AC002070.1, AC135178.2, AL590822.3, and AL355922.1 in cancer have not been reported.

Although the risk group has important value for tumor prognosis and systemic treatment, it cannot identify hot and cold tumors. The infiltration of immune cells in cold and hot tumors is different, and the efficacy of immunotherapy is also different. Therefore, it is crucial to distinguish between cold tumors and hot tumors and convert the former into the latter. It has been reported that different clusters are related to different tumor immune microenvironments and can distinguish hot and cold tumors (29–32). Therefore, we divided the patients into 2 clusters based on the R package CC, and the results indicated that the higher CD8 + T immune cells infiltration of cluster 1. Previous studies have shown that CD8 + T cells are the main driver of anti-tumor immunity (42). Additionally, the most prevalent stromal cell type in TME, cancer-associated fibroblasts, promote immunosuppression and support an environment that encourages tumor growth as important elements of anti-tumor immunity (43, 44). Our results show that cluster 2 Cancer-associated fibroblast is higher. So, we speculate that c1 is a hot tumor. Previous studies have shown that immunotherapy is effective against hot tumors but ineffective against cold tumors (45, 46). Therefore, patients with cluster 1 may have better efficacy in immunotherapy. This shows that our classification can guide personalized treatment, based on this lncRNA as a liquid biopsy, can be more convenient and effective to distinguish between hot and cold tumors.

At present, renal CT and B-ultrasound are the most commonly used auxiliary examination methods for the diagnosis of renal cell carcinoma in China. RCC lacks obvious clinical symptoms in the early stage, and there are no typical tumor markers for early diagnosis. Some patients with RCC are in the late stage of RCC at the first visit, unable to undergo surgery, and have a poor prognosis. At present, receptor tyrosine kinase inhibitors represented by sunitinib have been widely used in clinical practice. However, data analysis of 1059 patients showed that about 15% of patients with advanced renal cell carcinoma were congenitally resistant to targeted drugs, and the remaining patients often developed resistance after 6 to 15 months of sunitinib treatment (47). Qu et al. showed that LncARSR transmitted by exosomes competitively binds to miR-34/miR-449 and leads to the formation of sunitinib resistance (48). Therefore, it is particularly important to study lncRNA in-depth and find drug-sensitive markers for renal clear cell carcinoma. Our results showed that high-risk groups were more sensitive to Axitinib, Pazopanib, and Sunitinib. In summary, we established a large scoring model to provide a reference for drug selection in patients with renal clear cell carcinoma.

Although our risk model showed promising potential in terms of predictive power and distinguishing hot and cold tumors, it was important to note that this study also had limitations that need to be considered. First, the data of this study are from public sources, lacking evidence for further *in vivo* and *in vitro* experiments. Secondly, we only verify internally, like He et al. (32). We tried to download the information of patients with ccRCC from the International Cancer Genome Consortium or GEO database, but due to the lack of lncRNA expression data in these databases, we could not calculate the corresponding risk score.

## Conclusion

We established a prognostic model based on lncRNAs of 12 anoikis-related genes that can be used as prognostic markers for patients and guide targeted immunotherapy. In addition, the established classification can distinguish hot and cold tumors. This provides some reference for the clinical treatment of patients with ccRCC.

## Data availability statement

The original contributions presented in the study are included in the article/**Supplementary Material**. Further inquiries can be directed to the corresponding authors.

## Author contributions

RL, CH, ZL, TW, and TQ designed this study. RL, TQ, and CH collected the data. RL, CH, and ZL analyzed the data. RL, JS, KH, ZL, TW and CH wrote the main manuscript text. JS, TW and TQ revised the manuscript. All authors reviewed the manuscript.
