# Supplementary material for: Predicting prognosis, immunotherapy and distinguishing cold and hot tumors in clear cell renal cell carcinoma based on anoikis-related lncRNAs
Source: Front Immunol. 2023 Jun 9;14:1145450. doi: 10.3389/fimmu.2023.1145450 (PMC10288194; doi:10.3389/fimmu.2023.1145450)
Supplement: Supplementary file 1 [file DataSheet_1.pdf]

Supplementary Material

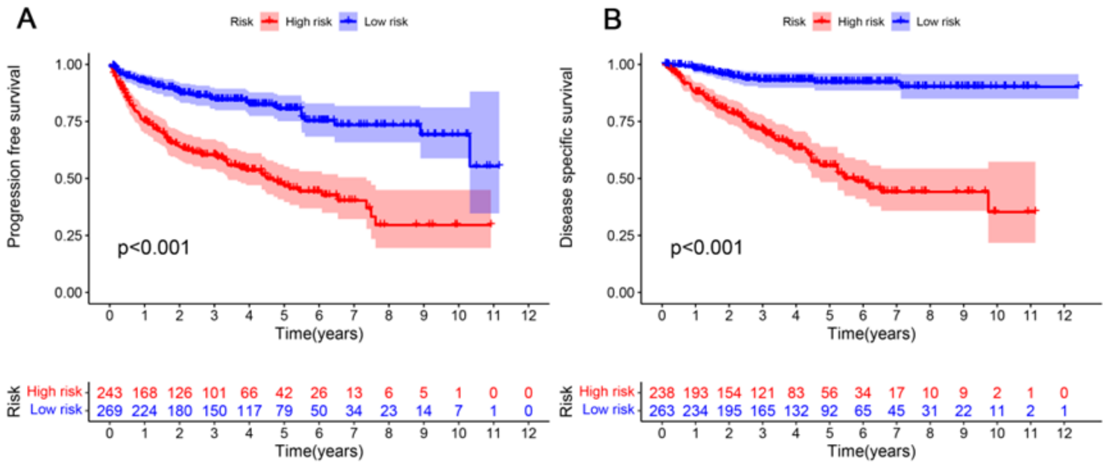

Supplementary Figure 1: The survival analysis of the disease-specific survival (DSS) and progression-free survival (PFS) on the TCGA set(A-B).

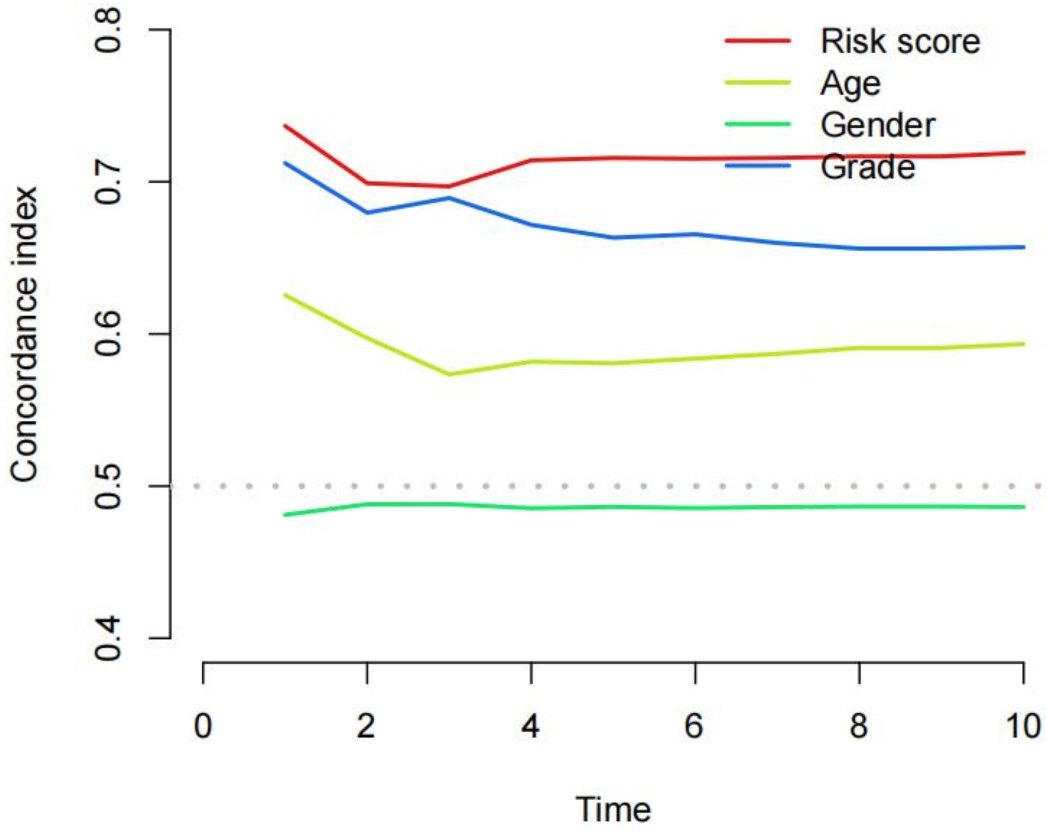

Supplementary Figure 2: C-index of risk score, age, gender stage.

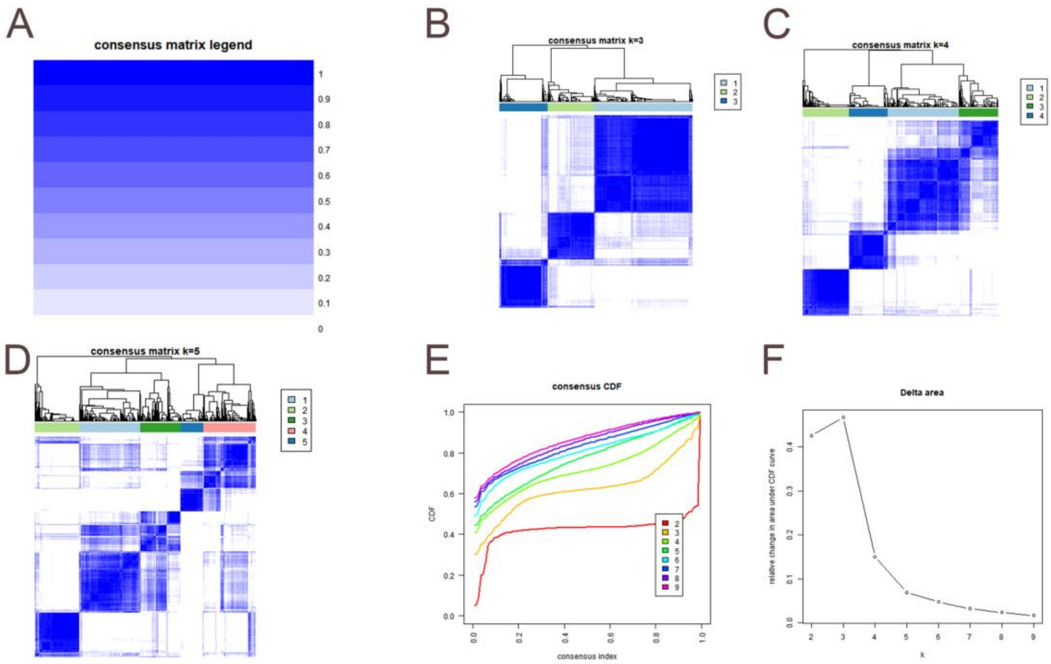

Supplementary Figure 3: Unsupervised consensus clustering of anoikis-related lncRNA in ccRCC patients. (A-D) Consensus matrices of the training cohort for K = 1, 3,4,5. (E) Cumulative distribution function (CDF). (F) Relative change of area under CDF curve.
