# Supplementary material for: Predicting prognosis, immunotherapy and distinguishing cold and hot tumors in clear cell renal cell carcinoma based on anoikis-related lncRNAs
Source: Front Immunol. 2023 Jun 9;14:1145450. doi: 10.3389/fimmu.2023.1145450 (PMC10288194; doi:10.3389/fimmu.2023.1145450)
Supplement: Supplementary file 2 [file Table_1.docx]

Tab**Supplementary Table 1:** The characteristics of the 512 ccRCC patients included in this study.

| characteristics |  | Total | Training set | Testing set | Pvalue |
| --- | --- | --- | --- | --- | --- |
| Age | <=65 | 339(66.21%) | 161(62.89%) | 178(69.53%) | 0.135 |
|  | >65 | 173(33.79%) | 95(37.11%) | 78(30.47%) |  |
| Gender | FEMALE | 117(26.35%) | 96(37.50%) | 21(0.82%) | 0.193 |
|  | MALE | 335(73.65%) | 160(62.50%) | 175(68.36%) |  |
| Grade | Grade I | 12(2.34%) | 6(2.34%) | 6(2.34%) | 0.334 |
|  | Grade II | 218(42.58%) | 103(40.23%) | 115(44.92%) |  |
|  | Grade III | 201(39.26%) | 105(41.02%) | 96(37.50%) |  |
|  | Grade IV | 73(14.26%) | 37(14.45%) | 36(14.06%) |  |
|  | Grade X | 5(0.98%) | 4(1.56%) | 1(0.39%) |  |
|  | unknow | 3(0.59%) | 1(0.39%) | 2(0.78%) |  |
| Stage | Stage I | 256(50.00%) | 126(49.22%) | 130(50.78%) | 0.954 |
|  | Stage II | 56(10.94%) | 32(12.50%) | 24(9.38%) |  |
|  | Stage III | 117(22.85%) | 57(22.27%) | 60(23.44%) |  |
|  | Stage IV | 83(16.21%) | 41(16.02%) | 42(16.41%) |  |
| T | T1 | 262(51.17%) | 129(50.39%) | 133(51.95%) | 0.976 |
|  | T2 | 68(13.28%) | 38(14.84%) | 30(11.72%) |  |
|  | T3 | 171(33.40%) | 84(32.81%) | 87(33.98%) |  |
|  | T4 | 11(2.15%) | 5(1.95%) | 6(2.34%) |  |
| M | M0 | 407(79.49%) | 208(81.25%) | 199(77.73%) | 0.300 |
|  | M1 | 78(15.23%) | 37(14.45%) | 41(16.02%) |  |
|  | MX | 25(4.88%) | 10(3.91%) | 15(5.86%) |  |
|  | unknow | 2(0.39%) | 1(0.39%) | 1(0.39%) |  |
| N | N0 | 229(44.73%) | 116(45.31%) | 113(44.14%) | 0.901 |
|  | N1 | 15(2.93%) | 6(2.34%) | 9(3.52%) |  |
|  | unknow | 268(52.34%) | 134(52.34%) | 134(52.34%) |  |
