# Supplementary material for: Predicting prognosis, immunotherapy and distinguishing cold and hot tumors in clear cell renal cell carcinoma based on anoikis-related lncRNAs
Source: Front Immunol. 2023 Jun 9;14:1145450. doi: 10.3389/fimmu.2023.1145450 (PMC10288194; doi:10.3389/fimmu.2023.1145450)
Supplement: Supplementary file 4 [file Table_3.docx]

**Supplementary table 3:** 233 differentially expressed genes.

| **gene** | **conMean** | **treatMean** | **logFC** | **pValue** | **fdr** |
| --- | --- | --- | --- | --- | --- |
| NTRK2 | 8.100245833 | 1.971820148 | -2.03843773 | 1.89E-28 | 9.66E-28 |
| CAV1 | 16.35719722 | 75.76094325 | 2.211528727 | 4.65E-35 | 6.37E-34 |
| EGFR | 10.89674444 | 30.45524177 | 1.482793384 | 2.82E-24 | 1.08E-23 |
| BCL2 | 5.542602778 | 9.679599815 | 0.804383786 | 3.14E-12 | 6.25E-12 |
| CASP8 | 3.215354167 | 5.46911793 | 0.766330514 | 2.18E-27 | 1.05E-26 |
| PTRH2 | 2.049858333 | 3.341983364 | 0.705180344 | 9.84E-30 | 5.83E-29 |
| BMF | 4.805151389 | 3.097316451 | -0.63356309 | 4.44E-09 | 7.50E-09 |
| ITGA5 | 7.957568056 | 31.08225009 | 1.965691451 | 5.25E-32 | 4.27E-31 |
| CDH1 | 50.49819167 | 18.26909464 | -1.46682659 | 1.45E-30 | 9.96E-30 |
| FN1 | 34.81148056 | 120.5603414 | 1.792120328 | 4.46E-22 | 1.51E-21 |
| PTGS2 | 6.033915278 | 2.658026248 | -1.18273909 | 2.50E-14 | 5.31E-14 |
| BAX | 9.073152778 | 21.48852569 | 1.243890647 | 8.34E-36 | 1.54E-34 |
| ERBB2 | 36.24516806 | 12.98270351 | -1.48119784 | 3.16E-37 | 1.15E-35 |
| ANGPTL4 | 11.70063056 | 361.1417926 | 4.947907199 | 1.49E-36 | 3.86E-35 |
| CYCS | 37.77281389 | 21.16936876 | -0.83537001 | 6.40E-28 | 3.19E-27 |
| ITGA2 | 12.14169028 | 6.699609427 | -0.85782038 | 3.13E-20 | 9.61E-20 |
| BIRC5 | 0.4219625 | 2.305272089 | 2.449750344 | 8.27E-34 | 8.44E-33 |
| MTOR | 9.662640278 | 4.355142144 | -1.14969765 | 9.06E-37 | 2.65E-35 |
| TIMP1 | 82.12318472 | 329.5530963 | 2.004649444 | 4.10E-26 | 1.80E-25 |
| BDNF | 0.118473611 | 0.524719039 | 2.146979389 | 5.05E-13 | 1.04E-12 |
| CSPG4 | 2.842113889 | 19.29812144 | 2.763424144 | 1.77E-33 | 1.73E-32 |
| ILK | 0.101161111 | 0.256596673 | 1.342847676 | 1.52E-16 | 3.64E-16 |
| HIF1A | 72.23023194 | 32.10889187 | -1.16962993 | 7.58E-23 | 2.60E-22 |
| ITGA3 | 25.83573472 | 38.96186506 | 0.592694827 | 1.18E-06 | 1.75E-06 |
| CCND1 | 32.41388333 | 158.4867669 | 2.289678608 | 2.61E-32 | 2.16E-31 |
| ATF4 | 48.44699167 | 94.46640591 | 0.963394286 | 4.90E-30 | 3.07E-29 |
| PLAUR | 1.992397222 | 5.749944177 | 1.529042645 | 2.10E-21 | 6.83E-21 |
| SKP2 | 0.444247222 | 0.773591312 | 0.800208838 | 1.47E-21 | 4.87E-21 |
| CHEK2 | 0.68115 | 1.597656192 | 1.229912538 | 3.26E-28 | 1.64E-27 |
| E2F1 | 0.601529167 | 2.676226802 | 2.153493788 | 5.07E-35 | 6.75E-34 |
| EGF | 65.15416389 | 1.405043068 | -5.53517112 | 4.00E-38 | 2.51E-36 |
| PIK3CG | 0.92305 | 2.511084104 | 1.443829646 | 1.75E-24 | 6.80E-24 |
| ITGB4 | 6.520554167 | 10.20714787 | 0.646513312 | 0.00281993 | 0.00346765 |
| CXCL12 | 39.13196528 | 16.95374455 | -1.20674361 | 7.61E-22 | 2.55E-21 |
| LGALS3 | 46.50495278 | 114.1029205 | 1.294879442 | 1.08E-16 | 2.63E-16 |
| ABHD4 | 17.67767222 | 10.96834677 | -0.68858223 | 8.23E-29 | 4.41E-28 |
| CD44 | 7.322744444 | 19.00162237 | 1.375666248 | 1.02E-18 | 2.91E-18 |
| ITGA4 | 1.182563889 | 4.253771534 | 1.846824421 | 1.58E-28 | 8.15E-28 |
| TGFB1 | 15.40386389 | 45.79667116 | 1.571950456 | 6.36E-32 | 4.99E-31 |
| HMCN1 | 0.889429167 | 2.152858226 | 1.275301695 | 9.74E-16 | 2.19E-15 |
| CEBPB | 10.50828194 | 26.9226281 | 1.357292433 | 1.54E-18 | 4.34E-18 |
| CDKN3 | 0.436854167 | 1.600842144 | 1.873607398 | 1.70E-31 | 1.24E-30 |
| CASP9 | 4.647808333 | 3.027142329 | -0.61859407 | 2.09E-26 | 9.35E-26 |
| PRKCA | 8.806220833 | 3.851482255 | -1.19310924 | 8.53E-32 | 6.46E-31 |
| TNFRSF10B | 7.785444444 | 22.2893183 | 1.517501188 | 3.72E-37 | 1.25E-35 |
| CDKN2A | 0.060926389 | 1.868556007 | 4.938712763 | 6.39E-42 | 1.40E-39 |
| CPT1A | 32.65847639 | 19.56442255 | -0.73922496 | 7.95E-21 | 2.53E-20 |
| CDKN1A | 49.52570694 | 77.50761922 | 0.64616057 | 1.06E-11 | 2.03E-11 |
| NTRK1 | 0.241308333 | 0.104119593 | -1.21263616 | 3.90E-16 | 9.10E-16 |
| PLAU | 56.30604444 | 12.68831756 | -2.14978902 | 1.02E-24 | 4.03E-24 |
| MYC | 6.944705556 | 20.36757043 | 1.552288464 | 6.63E-24 | 2.47E-23 |
| PLK1 | 0.260693056 | 1.068491682 | 2.03515162 | 1.88E-32 | 1.61E-31 |
| MUC1 | 56.218225 | 18.33169131 | -1.61669801 | 1.11E-31 | 8.23E-31 |
| LGALS1 | 64.22217917 | 276.1070024 | 2.104083953 | 3.33E-34 | 3.48E-33 |
| PYCARD | 1.865036111 | 7.632150277 | 2.032886014 | 2.37E-30 | 1.53E-29 |
| ITGB3 | 7.386413889 | 4.629977264 | -0.673869 | 1.21E-11 | 2.31E-11 |
| BID | 2.918027778 | 6.276572458 | 1.104983323 | 8.41E-36 | 1.54E-34 |
| HRAS | 10.31515139 | 16.19679094 | 0.650943007 | 3.17E-17 | 7.86E-17 |
| CDK11A | 0.103084722 | 0.197439372 | 0.937579175 | 8.49E-11 | 1.55E-10 |
| CCR7 | 0.551027778 | 1.751636229 | 1.668506241 | 7.80E-20 | 2.38E-19 |
| GRHL2 | 4.818829167 | 0.345096118 | -3.80361251 | 1.09E-36 | 2.99E-35 |
| BIRC3 | 5.2195875 | 26.39130721 | 2.33805511 | 1.83E-29 | 1.00E-28 |
| NOTCH1 | 5.328443056 | 8.719347135 | 0.71050607 | 5.11E-12 | 1.01E-11 |
| RHOG | 16.61494444 | 33.10735952 | 0.994670484 | 7.59E-32 | 5.84E-31 |
| MMP13 | 0.0158125 | 0.46956451 | 4.892185988 | 2.48E-05 | 3.40E-05 |
| FAS | 2.6950625 | 6.486196118 | 1.267053916 | 1.50E-27 | 7.30E-27 |
| MTA1 | 2.5254875 | 4.417835305 | 0.806777736 | 7.27E-18 | 1.89E-17 |
| EDA2R | 1.157708333 | 5.740215527 | 2.309833072 | 2.80E-37 | 1.12E-35 |
| CCN6 | 0.791369444 | 0.513739002 | -0.62331576 | 1.45E-28 | 7.57E-28 |
| MMP9 | 0.668116667 | 10.75029723 | 4.00813269 | 6.31E-25 | 2.54E-24 |
| MAPK11 | 2.968063889 | 6.868787431 | 1.210533291 | 1.27E-26 | 5.76E-26 |
| SOD2 | 43.58383611 | 99.48949538 | 1.190751023 | 3.45E-15 | 7.49E-15 |
| PTHLH | 0.239345833 | 11.28470665 | 5.559128415 | 6.58E-36 | 1.31E-34 |
| GLI2 | 0.326593056 | 0.656235305 | 1.006719097 | 1.93E-09 | 3.32E-09 |
| EZH2 | 0.532166667 | 1.973430684 | 1.890755794 | 3.82E-36 | 8.38E-35 |
| CXCR4 | 14.5266375 | 101.3142697 | 2.802064682 | 3.91E-39 | 3.43E-37 |
| TNFSF10 | 55.26217917 | 89.12315823 | 0.689507904 | 4.02E-08 | 6.46E-08 |
| ANGPTL2 | 11.11773611 | 29.37134159 | 1.401546121 | 6.56E-18 | 1.72E-17 |
| ETV4 | 1.0683375 | 0.4325122 | -1.30455475 | 6.09E-26 | 2.62E-25 |
| HTRA1 | 78.89351389 | 130.4282359 | 0.725277625 | 2.65E-09 | 4.51E-09 |
| CEACAM3 | 0.029338889 | 0.103003512 | 1.811807389 | 1.89E-16 | 4.46E-16 |
| LAMC2 | 9.623940278 | 3.196492791 | -1.59013785 | 2.76E-22 | 9.41E-22 |
| LAMA3 | 1.143919444 | 1.976342329 | 0.788847403 | 2.59E-05 | 3.54E-05 |
| CDH2 | 4.889347222 | 11.75283087 | 1.265294528 | 1.41E-23 | 5.22E-23 |
| EDIL3 | 10.986 | 24.14634806 | 1.136138813 | 1.66E-09 | 2.89E-09 |
| ZEB2 | 2.745876389 | 4.539480776 | 0.725260611 | 7.23E-16 | 1.64E-15 |
| OLFM3 | 0.26305 | 0.006698891 | -5.29527097 | 7.10E-48 | 3.12E-45 |
| CLU | 204.9160347 | 319.3917427 | 0.640294135 | 2.43E-05 | 3.34E-05 |
| SPINK1 | 13.6831625 | 6.497848983 | -1.07436759 | 1.19E-15 | 2.64E-15 |
| CPEB2 | 7.732884722 | 13.01309667 | 0.750885704 | 2.41E-15 | 5.29E-15 |
| SERPINA1 | 112.0290181 | 352.1607303 | 1.652361571 | 7.21E-14 | 1.51E-13 |
| TNFRSF1A | 23.57652639 | 46.08548115 | 0.966961136 | 2.83E-27 | 1.35E-26 |
| FASLG | 0.154502778 | 1.806089834 | 3.547164972 | 4.00E-35 | 5.85E-34 |
| AFP | 0.790455556 | 0.158992791 | -2.31372299 | 4.52E-09 | 7.57E-09 |
| NOX4 | 14.67691806 | 6.187868946 | -1.24603451 | 0.00338911 | 0.00414434 |
| PBK | 0.402554167 | 1.394189834 | 1.792172186 | 2.21E-30 | 1.45E-29 |
| CD63 | 192.9466833 | 351.6060693 | 0.865757729 | 3.92E-31 | 2.82E-30 |
| LTB4R2 | 0.072877778 | 0.399562847 | 2.454871567 | 5.38E-32 | 4.29E-31 |
| HRC | 2.228811111 | 3.445313678 | 0.628360984 | 9.32E-05 | 0.00012594 |
| CCN2 | 200.7463431 | 88.46157357 | -1.1822509 | 1.41E-19 | 4.21E-19 |
| RHOB | 199.9255333 | 374.7507516 | 0.906468635 | 1.30E-09 | 2.27E-09 |
| PLG | 28.52377361 | 3.239898336 | -3.13814441 | 2.12E-18 | 5.86E-18 |
| MET | 21.37009861 | 47.62502865 | 1.156126395 | 2.59E-24 | 9.99E-24 |
| RAF1 | 7.5881125 | 4.414395749 | -0.7815251 | 7.31E-33 | 6.69E-32 |
| PRKCQ | 8.924015278 | 2.802409427 | -1.67102524 | 5.71E-26 | 2.48E-25 |
| BRCA2 | 0.132501389 | 0.447858965 | 1.757037003 | 1.92E-32 | 1.62E-31 |
| HAVCR2 | 7.930748611 | 29.41661331 | 1.891102202 | 1.18E-18 | 3.37E-18 |
| DOCK1 | 16.401025 | 10.58201904 | -0.63217106 | 2.81E-18 | 7.56E-18 |
| INHBB | 1.879383333 | 26.59988078 | 3.823088514 | 4.76E-38 | 2.61E-36 |
| KDR | 19.35559028 | 44.87117579 | 1.213038683 | 1.51E-12 | 3.06E-12 |
| MDM2 | 4.1923875 | 7.572604067 | 0.853017429 | 1.27E-26 | 5.76E-26 |
| ZEB1 | 4.683423611 | 7.647538447 | 0.707431921 | 2.98E-11 | 5.60E-11 |
| KL | 56.1441125 | 19.65834917 | -1.51399257 | 3.00E-18 | 8.03E-18 |
| PRKCI | 11.00514583 | 6.935574122 | -0.66609105 | 1.14E-21 | 3.81E-21 |
| CRYAB | 121.575125 | 245.6114196 | 1.014529565 | 3.90E-12 | 7.72E-12 |
| HK2 | 1.712736111 | 18.42961793 | 3.427651371 | 2.24E-36 | 5.18E-35 |
| LTF | 40.47107917 | 22.75097116 | -0.83096319 | 1.25E-12 | 2.56E-12 |
| MGAT5 | 18.19790278 | 9.684549538 | -0.91001535 | 1.20E-27 | 5.94E-27 |
| SPIB | 0.093793056 | 0.240466728 | 1.358284277 | 3.94E-11 | 7.33E-11 |
| TRIM31 | 0.047193056 | 0.098415157 | 1.060305942 | 4.83E-07 | 7.32E-07 |
| PDGFRB | 20.65581528 | 41.19622736 | 0.995964221 | 2.84E-12 | 5.67E-12 |
| PLAT | 23.40118194 | 7.030693346 | -1.73484252 | 2.67E-35 | 4.03E-34 |
| TLR3 | 5.839425 | 16.87262994 | 1.530786644 | 3.19E-24 | 1.21E-23 |
| VEGFA | 10.41716389 | 124.0674177 | 3.574089831 | 1.51E-37 | 6.63E-36 |
| IL1RAP | 0.830041667 | 1.700614603 | 1.034800567 | 1.13E-15 | 2.53E-15 |
| UBE2C | 0.868522222 | 6.41152329 | 2.884032495 | 6.78E-35 | 8.51E-34 |
| TWIST1 | 0.302070833 | 0.690931608 | 1.193656022 | 9.67E-09 | 1.61E-08 |
| BMP6 | 12.46189722 | 2.800957671 | -2.15353164 | 2.18E-19 | 6.41E-19 |
| BNIP3L | 37.83830417 | 71.29778078 | 0.91400974 | 1.00E-26 | 4.64E-26 |
| KDM3A | 3.802126389 | 6.957721072 | 0.871808354 | 1.70E-29 | 9.42E-29 |
| PRDX4 | 21.40623194 | 56.92219372 | 1.410960397 | 4.46E-36 | 9.33E-35 |
| BNIP3 | 17.59959306 | 52.41795453 | 1.574518987 | 3.57E-30 | 2.27E-29 |
| LMO3 | 1.6038125 | 0.155168946 | -3.36959372 | 7.96E-38 | 3.88E-36 |
| TUBB3 | 0.014091667 | 0.066586322 | 2.240383591 | 2.16E-13 | 4.47E-13 |
| SLC2A1 | 12.69474444 | 50.19007264 | 1.983170682 | 5.31E-30 | 3.29E-29 |
| HMOX1 | 27.17382222 | 141.2829627 | 2.378298089 | 1.58E-32 | 1.39E-31 |
| CD36 | 3.497726389 | 16.14532311 | 2.20662697 | 2.19E-23 | 7.89E-23 |
| PIK3R2 | 0.006156944 | 0.030226617 | 2.295533082 | 1.47E-17 | 3.73E-17 |
| CDH3 | 4.518719444 | 0.963728281 | -2.22921564 | 1.18E-35 | 2.00E-34 |
| EEF2K | 3.175666667 | 5.568378004 | 0.810197662 | 6.48E-25 | 2.58E-24 |
| LRP1 | 10.17482361 | 19.1641597 | 0.913406957 | 2.04E-17 | 5.16E-17 |
| PTK6 | 0.502348611 | 0.955513309 | 0.92758708 | 5.43E-05 | 7.36E-05 |
| LPAR1 | 4.820743056 | 2.782883919 | -0.79267481 | 1.96E-12 | 3.94E-12 |
| CEACAM1 | 8.492922222 | 3.931786137 | -1.11107619 | 1.20E-24 | 4.71E-24 |
| SIRPA | 16.69714167 | 48.75993031 | 1.546094911 | 8.67E-33 | 7.77E-32 |
| TRAF2 | 3.042058333 | 6.283720887 | 1.046571282 | 2.29E-33 | 2.18E-32 |
| ADCY10 | 0.038923611 | 0.152131238 | 1.966598958 | 1.89E-19 | 5.60E-19 |
| TNFRSF12A | 33.50888472 | 54.35522514 | 0.697875057 | 3.01E-06 | 4.37E-06 |
| APOBEC3G | 1.202336111 | 6.14521756 | 2.353623832 | 1.77E-35 | 2.88E-34 |
| BAG1 | 21.32002778 | 8.731464695 | -1.28791373 | 1.11E-34 | 1.28E-33 |
| COL13A1 | 0.198543056 | 0.334977634 | 0.754612871 | 0.00225807 | 0.00280026 |
| MNX1 | 0.028354167 | 0.151827911 | 2.420804372 | 3.79E-07 | 5.78E-07 |
| RAD9A | 1.783848611 | 3.162337893 | 0.825998342 | 1.11E-16 | 2.68E-16 |
| IFI27 | 13.16738472 | 35.73754399 | 1.440471662 | 1.92E-21 | 6.30E-21 |
| MEGF11 | 0.433215278 | 0.908884843 | 1.069013392 | 1.19E-07 | 1.87E-07 |
| ITPRIP | 6.579540278 | 12.71202717 | 0.950135424 | 9.85E-16 | 2.21E-15 |
| BCL2L15 | 0.590498611 | 0.170531608 | -1.7918945 | 6.46E-12 | 1.25E-11 |
| NOTCH3 | 15.37742361 | 39.41154436 | 1.357804474 | 7.49E-19 | 2.16E-18 |
| TPM1 | 16.4573875 | 31.72047024 | 0.946678823 | 8.65E-20 | 2.60E-19 |
| COL4A2 | 22.59543333 | 65.29636081 | 1.530971363 | 3.39E-26 | 1.50E-25 |
| CD151 | 54.47230417 | 87.67152274 | 0.686585412 | 3.19E-18 | 8.49E-18 |
| MMP11 | 1.375623611 | 5.498199076 | 1.998873361 | 1.26E-29 | 7.30E-29 |
| ARHGEF7 | 5.031388889 | 8.228868762 | 0.709737411 | 4.75E-24 | 1.78E-23 |
| BST2 | 58.50799306 | 145.0409468 | 1.309754611 | 6.49E-23 | 2.26E-22 |
| CCDC178 | 1.070848611 | 0.388681331 | -1.46209482 | 1.41E-29 | 7.93E-29 |
| SHC1 | 13.59778194 | 28.06550203 | 1.045426527 | 9.45E-30 | 5.68E-29 |
| BUB1 | 0.252323611 | 1.089992052 | 2.110970497 | 3.76E-33 | 3.51E-32 |
| CDC25C | 0.066197222 | 0.435677449 | 2.718417856 | 1.82E-34 | 2.00E-33 |
| CDK1 | 1.564309722 | 2.481084473 | 0.665444671 | 5.15E-11 | 9.45E-11 |
| SETD2 | 9.415972222 | 5.798361738 | -0.69946472 | 1.74E-25 | 7.29E-25 |
| TP73 | 0.029381944 | 0.243811091 | 3.052761974 | 9.89E-35 | 1.17E-33 |
| TDGF1 | 8.590694444 | 0.781859889 | -3.45779276 | 5.90E-37 | 1.85E-35 |
| EDAR | 0.579269444 | 0.267392606 | -1.11527499 | 4.00E-19 | 1.17E-18 |
| MAD2L1 | 0.764773611 | 1.444445656 | 0.917411279 | 9.65E-21 | 3.03E-20 |
| PDCD6IP | 23.75839722 | 13.89519464 | -0.77385147 | 2.82E-34 | 3.02E-33 |
| DYNLL2 | 38.16250694 | 19.1583122 | -0.99418548 | 2.92E-38 | 2.14E-36 |
| BAG4 | 7.604819444 | 5.048626802 | -0.59102296 | 1.37E-29 | 7.84E-29 |
| F3 | 8.098391667 | 4.177239926 | -0.95508541 | 2.53E-23 | 9.03E-23 |
| ADAMTSL1 | 1.410168056 | 0.781096673 | -0.85229408 | 4.95E-23 | 1.74E-22 |
| SERPINB1 | 20.17132361 | 33.1881146 | 0.718360919 | 4.63E-27 | 2.18E-26 |
| CEACAM4 | 0.157111111 | 0.57287098 | 1.866425042 | 3.61E-21 | 1.16E-20 |
| CRYBA1 | 0.029270833 | 0.105528281 | 1.85009396 | 2.73E-10 | 4.94E-10 |
| SERPINE1 | 28.47536389 | 130.4732895 | 2.195968305 | 1.28E-15 | 2.82E-15 |
| NTRK3 | 0.510222222 | 0.290238262 | -0.81388801 | 2.44E-08 | 3.95E-08 |
| TNC | 32.46362639 | 15.37210961 | -1.078509 | 1.18E-06 | 1.74E-06 |
| IRF6 | 17.07551111 | 5.541577449 | -1.62356015 | 1.24E-34 | 1.40E-33 |
| XAF1 | 0.527386111 | 2.266666913 | 2.10364092 | 2.12E-25 | 8.80E-25 |
| SFRP1 | 105.5623833 | 2.852728281 | -5.20960968 | 1.10E-40 | 1.21E-38 |
| CSK | 7.061911111 | 13.78842717 | 0.965327333 | 9.17E-34 | 9.15E-33 |
| ENDOG | 1.874491667 | 1.198813494 | -0.64489218 | 5.41E-10 | 9.65E-10 |
| FOXC2 | 1.839848611 | 4.4705939 | 1.280879439 | 6.30E-12 | 1.23E-11 |
| RACK1 | 131.5534472 | 302.4846098 | 1.201212688 | 2.17E-35 | 3.41E-34 |
| ARHGDIB | 43.85409444 | 139.2813007 | 1.66721813 | 4.37E-35 | 6.19E-34 |
| PRKD1 | 8.9714625 | 4.390664695 | -1.03090382 | 4.62E-31 | 3.27E-30 |
| LDHA | 97.85286944 | 309.045141 | 1.65913152 | 9.22E-35 | 1.12E-33 |
| ANXA2 | 27.4178375 | 46.5747549 | 0.76443339 | 8.87E-15 | 1.92E-14 |
| QSOX1 | 14.71990972 | 24.33708558 | 0.725387589 | 1.41E-05 | 1.99E-05 |
| MAOA | 36.6270375 | 21.42669649 | -0.77349958 | 1.51E-18 | 4.28E-18 |
| JUP | 99.25651944 | 48.50377763 | -1.03306475 | 2.15E-30 | 1.43E-29 |
| NDRG1 | 79.61540278 | 207.6457285 | 1.38300472 | 9.81E-27 | 4.58E-26 |
| OCLN | 5.338586111 | 1.272531793 | -2.068756 | 1.01E-35 | 1.78E-34 |
| CEACAM8 | 0.008329167 | 0.023567652 | 1.500563997 | 0.01677344 | 0.01979446 |
| PITPNC1 | 3.635616667 | 7.344853604 | 1.014533643 | 7.06E-23 | 2.44E-22 |
| AFAP1L1 | 2.762527778 | 8.181623105 | 1.566398108 | 4.83E-29 | 2.62E-28 |
| INSR | 15.49788056 | 38.71499945 | 1.320821691 | 1.54E-25 | 6.48E-25 |
| HSPB1 | 190.6931333 | 324.2952303 | 0.76605491 | 8.85E-10 | 1.57E-09 |
| NGF | 1.649326389 | 7.307201664 | 2.147442098 | 4.79E-25 | 1.96E-24 |
| TP63 | 0.269508333 | 0.12081756 | -1.15749973 | 2.69E-06 | 3.92E-06 |
| KRT14 | 0.105555556 | 0.335135305 | 1.666741164 | 0.00014508 | 0.00019418 |
| SPHK1 | 0.6413875 | 2.047613678 | 1.674675406 | 4.30E-19 | 1.25E-18 |
| RAC3 | 3.092483333 | 1.99628817 | -0.63144583 | 8.80E-18 | 2.25E-17 |
| LCK | 2.160191667 | 5.263756192 | 1.284933344 | 6.69E-16 | 1.54E-15 |
| UCHL1 | 37.18446528 | 9.471375231 | -1.9730542 | 6.87E-31 | 4.79E-30 |
| BRCA1 | 0.504186111 | 0.877453789 | 0.799366772 | 8.35E-20 | 2.53E-19 |
| NOS2 | 0.591722222 | 1.204818484 | 1.025823827 | 0.00192335 | 0.00240556 |
| FYN | 5.440455556 | 10.61351257 | 0.964102833 | 1.71E-20 | 5.33E-20 |
| SNAI1 | 3.506938889 | 6.216434566 | 0.825875069 | 7.44E-06 | 1.05E-05 |
| C5AR1 | 3.773981944 | 9.837569871 | 1.382214457 | 2.19E-23 | 7.89E-23 |
| PRDM1 | 1.2449875 | 6.224492606 | 2.321824982 | 1.68E-36 | 4.10E-35 |
| SKI | 11.49599583 | 17.6057634 | 0.614916341 | 1.82E-16 | 4.32E-16 |
| CLDN18 | 0.119468056 | 0.220387985 | 0.883420666 | 0.0003322 | 0.00043794 |
| SPTA1 | 0.010941667 | 0.068878558 | 2.654222434 | 4.52E-23 | 1.60E-22 |
| CENPF | 0.377843056 | 1.32974732 | 1.815293116 | 2.03E-30 | 1.37E-29 |
| DOK2 | 1.503051389 | 5.613797782 | 1.901082761 | 1.24E-29 | 7.28E-29 |
| IKZF3 | 0.343601389 | 2.134966543 | 2.635405689 | 1.35E-28 | 7.12E-28 |
| SNCG | 10.42653056 | 20.23820333 | 0.95682204 | 3.78E-08 | 6.11E-08 |
| USP11 | 28.06661667 | 17.5693024 | -0.67579825 | 7.71E-26 | 3.29E-25 |
| SLPI | 176.4101194 | 51.17703494 | -1.78536485 | 1.76E-18 | 4.92E-18 |
| HTRA3 | 1.595480556 | 3.313628466 | 1.054420826 | 7.13E-08 | 1.13E-07 |
| EFHD2 | 11.36917917 | 30.8195475 | 1.438717582 | 6.16E-35 | 7.95E-34 |
| IRX1 | 7.207779167 | 0.307203327 | -4.55228906 | 9.31E-41 | 1.21E-38 |
| KIF18A | 0.223795833 | 0.657672089 | 1.555185268 | 7.94E-30 | 4.84E-29 |
| ZG16B | 0.124116667 | 0.268130314 | 1.11123748 | 1.51E-08 | 2.49E-08 |
