# Supplementary material for: Predicting prognosis, immunotherapy and distinguishing cold and hot tumors in clear cell renal cell carcinoma based on anoikis-related lncRNAs
Source: Front Immunol. 2023 Jun 9;14:1145450. doi: 10.3389/fimmu.2023.1145450 (PMC10288194; doi:10.3389/fimmu.2023.1145450)
Supplement: Supplementary file 5 [file Table_4.docx]

**Supplementary table 4:** 3973 ARlncRNAs were further screened using correlation coefficient > 0.4 and p value < 0.05

| ID |
| --- |
| DHRS4-AS1 |
| AC024588.1 |
| LINC00852 |
| AP002748.4 |
| PLS3-AS1 |
| AC073957.3 |
| AC098484.4 |
| AC090971.2 |
| AC012157.2 |
| RNF216P1 |
| AL139275.2 |
| AC009301.1 |
| AC139887.1 |
| AP003774.2 |
| IDH1-AS1 |
| LINC01786 |
| LINC02202 |
| UBOX5-AS1 |
| ASTN2-AS1 |
| AC005753.3 |
| PRR34-AS1 |
| AC020779.2 |
| AC010913.1 |
| LINC00513 |
| AC023158.2 |
| AC004241.3 |
| AC025031.3 |
| LINC02827 |
| REPIN1-AS1 |
| LINC01686 |
| AP002807.1 |
| AP006621.2 |
| AC131971.1 |
| AL360091.1 |
| C8orf44 |
| PARD3-AS1 |
| RAP2C-AS1 |
| AP001505.1 |
| RFX5-AS1 |
| LINC01842 |
| AC010761.6 |
| L3MBTL4-AS1 |
| PANK2-AS1 |
| AL031602.2 |
| AL096701.4 |
| AC104564.1 |
| AC010149.1 |
| AP000331.1 |
| IDH2-DT |
| CEP83-DT |
| N4BP2L2-IT2 |
| AC066613.1 |
| AC011471.2 |
| AC009053.3 |
| ZNF503-AS2 |
| LINC01176 |
| HEXD-IT1 |
| AC016876.3 |
| H1-10-AS1 |
| AC024941.2 |
| AL596442.4 |
| AC009509.4 |
| AL355297.3 |
| SIRLNT |
| AL354833.2 |
| LINC01816 |
| AL391056.1 |
| AL158832.1 |
| AC025287.3 |
| AC027601.5 |
| AC123595.2 |
| ZNF30-AS1 |
| TMEM72-AS1 |
| AC010503.4 |
| MALAT1 |
| AC005391.1 |
| AP003392.1 |
| LINC01711 |
| AC093297.2 |
| LINC02015 |
| DAPK1-IT1 |
| ARHGEF35-AS1 |
| AC090772.1 |
| AL512791.2 |
| RDH10-AS1 |
| LINC02275 |
| LINC01402 |
| AC007613.1 |
| AC006538.1 |
| AC084018.1 |
| AL596202.1 |
| AL158151.4 |
| AC015802.4 |
| AC139795.2 |
| AL136169.1 |
| TTC21B-AS1 |
| GRPEL2-AS1 |
| ASH1L-AS1 |
| AC073283.2 |
| AC023389.1 |
| C1orf195 |
| AC097641.2 |
| AC131009.3 |
| AC104458.1 |
| AL591845.1 |
| ENTPD1-AS1 |
| AL451064.1 |
| SNHG9 |
| LINC00324 |
| AC009560.1 |
| LINC02038 |
| GK-AS1 |
| AC092645.2 |
| SNHG3 |
| AC009570.1 |
| AC108159.1 |
| AP001596.1 |
| AC105916.1 |
| MAGI1-IT1 |
| AC100830.1 |
| AL035448.1 |
| AC004921.1 |
| AC090541.1 |
| AC009133.1 |
| AC002456.1 |
| AC009242.1 |
| CACNA1C-AS2 |
| AC005277.2 |
| AC022784.1 |
| AC026412.3 |
| MGC16275 |
| AC083843.2 |
| AL035587.1 |
| AC005363.2 |
| AC068790.4 |
| AL031985.3 |
| AC007998.3 |
| ITGB5-AS1 |
| LINC02798 |
| AC009084.1 |
| AC004148.1 |
| AC005006.1 |
| AL162171.2 |
| AC074135.1 |
| MROCKI |
| AP000695.2 |
| AP000808.1 |
| AL139022.1 |
| AP002381.2 |
| AL049840.3 |
| RUSC1-AS1 |
| LINC00926 |
| AL121672.1 |
| AL450043.1 |
| AFDN-DT |
| TSC22D1-AS1 |
| AC039056.2 |
| FAM160A1-DT |
| AP001922.6 |
| AC008870.2 |
| SCAT2 |
| AF124730.1 |
| AC016831.1 |
| NFYC-AS1 |
| WNT5A-AS1 |
| AC008760.1 |
| AL161457.2 |
| AC116407.1 |
| LINC02257 |
| AC106897.1 |
| AC022400.4 |
| AL391684.1 |
| AP001269.2 |
| LINC01277 |
| MKNK1-AS1 |
| AC002066.1 |
| AC013553.3 |
| SNHG17 |
| AL606834.1 |
| AL158166.1 |
| WWTR1-AS1 |
| AL031848.1 |
| LINC01679 |
| TMSB15B-AS1 |
| PDC-AS1 |
| AC104135.1 |
| AC007406.2 |
| SNHG12 |
| AC093535.1 |
| AC018638.6 |
| AL132656.2 |
| AC012358.1 |
| AL078600.1 |
| AC012404.1 |
| AC104532.2 |
| AC107021.1 |
| AC068620.2 |
| AL157392.2 |
| AL357079.1 |
| AL159972.1 |
| AC011933.3 |
| AL450326.1 |
| AC004253.1 |
| AC090061.1 |
| Z99572.1 |
| FAM198B-AS1 |
| AC010327.8 |
| AC079684.1 |
| AC005479.1 |
| AC016705.2 |
| AC009090.6 |
| AC124798.1 |
| AC079949.5 |
| AC034236.3 |
| AL096854.1 |
| LINC00539 |
| A2M-AS1 |
| AC116312.1 |
| AL021978.1 |
| BAALC-AS2 |
| AC097504.2 |
| AC079336.5 |
| AC005828.4 |
| LINC01150 |
| AL031710.1 |
| AP002813.1 |
| LINC00954 |
| LINC02147 |
| GHRLOS |
| AC005840.2 |
| LINC02340 |
| AC004923.4 |
| LACTB2-AS1 |
| AC010186.3 |
| AC012254.3 |
| AL683813.1 |
| AC100812.1 |
| AC133919.2 |
| ABCC5-AS1 |
| MIS18A-AS1 |
| VPS9D1-AS1 |
| BACH1-IT2 |
| ZNF793-AS1 |
| AP002985.1 |
| AC093599.2 |
| AC023669.1 |
| AC008083.3 |
| AC040160.1 |
| AC012676.4 |
| AC006017.1 |
| AP001160.4 |
| AL138756.1 |
| SPATA13-AS1 |
| AL354872.1 |
| CDC42-AS1 |
| AC087468.2 |
| AC093827.4 |
| AL023553.1 |
| AC105105.3 |
| NRAD1 |
| AC115102.1 |
| LINC01428 |
| AC090337.2 |
| AP005899.1 |
| AC016924.1 |
| AL442128.2 |
| AC007546.1 |
| AC015813.1 |
| AC015849.3 |
| AC048382.1 |
| LINC00115 |
| AC021491.4 |
| AL138787.2 |
| AC004803.1 |
| AC005387.1 |
| AC108519.1 |
| SRD5A3-AS1 |
| AC093281.2 |
| LIF-AS1 |
| AC009305.1 |
| AC025917.1 |
| AL049780.1 |
| AC027544.2 |
| AP003465.2 |
| AL356608.3 |
| CCDC13-AS1 |
| LINC01976 |
| AC138207.1 |
| LINC01543 |
| SMC5-AS1 |
| AL591767.1 |
| LINC01230 |
| AC093726.2 |
| AC005529.1 |
| AP000919.1 |
| AC127521.1 |
| LINC01012 |
| HOXA-AS2 |
| MIR100HG |
| AC020558.6 |
| AL132639.2 |
| ZNF433-AS1 |
| MHENCR |
| AC136424.2 |
| ZBTB40-IT1 |
| HOTAIRM1 |
| AC073655.2 |
| AC084782.3 |
| AL157935.1 |
| PLBD1-AS1 |
| AL354989.1 |
| AC011407.1 |
| CATIP-AS2 |
| AC008537.4 |
| PCAT7 |
| SIM1-AS1 |
| AC079906.1 |
| AL359962.3 |
| AC004596.1 |
| AL512274.1 |
| AL031432.4 |
| AC006449.5 |
| AC073569.1 |
| AC025442.2 |
| AL731567.1 |
| AL138895.1 |
| LINC01215 |
| LINC01352 |
| MAFG-DT |
| AC146507.3 |
| AP001469.3 |
| ZNF790-AS1 |
| AL049869.2 |
| DHDDS-AS1 |
| AL049629.1 |
| BACH1-AS1 |
| KLHL7-DT |
| AC005070.3 |
| AC026401.3 |
| AL365330.1 |
| MIR583HG |
| AC006369.1 |
| AC018529.1 |
| AP000223.1 |
| ADCY6-DT |
| LINC00342 |
| AC073517.1 |
| LINC00641 |
| AL731577.1 |
| LINC01751 |
| GTSE1-DT |
| SNHG20 |
| ELF3-AS1 |
| AP005136.4 |
| AL033519.5 |
| LINC01588 |
| AL021807.1 |
| AC011815.2 |
| AC087175.1 |
| AL161421.1 |
| AL117379.1 |
| AC021739.2 |
| AL358976.1 |
| AC104463.2 |
| AL021707.2 |
| AL133410.1 |
| LINC01238 |
| AP000759.1 |
| AL136040.1 |
| LINC01320 |
| FAM106A |
| ZNF571-AS1 |
| AKT3-IT1 |
| AL139246.4 |
| AC004584.1 |
| AC108102.1 |
| AC090229.1 |
| LMNTD2-AS1 |
| AP001094.2 |
| AC010173.1 |
| AL359513.1 |
| LINC02609 |
| PAXIP1-AS2 |
| AC090152.1 |
| AC008119.1 |
| EHMT2-AS1 |
| AC122129.1 |
| AC010530.1 |
| AL139351.3 |
| AL354892.3 |
| AC009486.1 |
| SCAMP1-AS1 |
| RFX3-AS1 |
| LINC00857 |
| AL390066.2 |
| PTCSC1 |
| AL118556.1 |
| AC046158.2 |
| LINC02709 |
| AC008875.2 |
| GAPLINC |
| AC092306.1 |
| AC025871.2 |
| LINC01060 |
| PCBP2-OT1 |
| CLDN10-AS1 |
| AC092422.1 |
| LINC01135 |
| AC006207.1 |
| AC087501.4 |
| AC099063.4 |
| AC130895.1 |
| AC027601.2 |
| AL450992.3 |
| AC022898.1 |
| AC018653.4 |
| AC106037.2 |
| AL592494.2 |
| AC005899.1 |
| AL031587.3 |
| AC097059.1 |
| AC103923.1 |
| AC078778.1 |
| AL513128.3 |
| Z93241.1 |
| AC067930.3 |
| AC100803.4 |
| AL021368.2 |
| AC074286.1 |
| AC093227.3 |
| AC132192.1 |
| LINC01474 |
| AP000787.1 |
| TBC1D22A-AS1 |
| CRTC3-AS1 |
| CEP250-AS1 |
| AL132642.1 |
| AC027796.1 |
| LINC02048 |
| AL022313.2 |
| AL355835.1 |
| LINC01144 |
| PSMA3-AS1 |
| AC005332.1 |
| AC010201.3 |
| AC098851.1 |
| AC026356.1 |
| AL135960.1 |
| AC104984.3 |
| MAP3K4-AS1 |
| AC005046.1 |
| AL162741.1 |
| AC012363.2 |
| FAM27E3 |
| AC008663.3 |
| FRG1-DT |
| AC004982.1 |
| AC073218.1 |
| AL031846.2 |
| LINC02586 |
| AC007637.1 |
| SLC25A30-AS1 |
| AC090948.3 |
| AP001626.1 |
| AC105206.2 |
| AC004908.3 |
| AC138150.2 |
| AC244093.3 |
| FSIP2-AS2 |
| AL732509.1 |
| AC091185.1 |
| ODC1-DT |
| C3orf35 |
| AC023908.3 |
| AC092718.5 |
| AC002116.2 |
| AC106881.1 |
| XIAP-AS1 |
| AC092755.2 |
| AC005014.4 |
| AL078605.1 |
| AP001021.2 |
| AC010618.3 |
| AL442067.3 |
| AC091563.1 |
| AC087501.1 |
| AC106845.1 |
| AC009955.4 |
| AL021707.3 |
| AP005203.1 |
| AL357874.1 |
| AC104211.1 |
| ZNF460-AS1 |
| AL359532.1 |
| AL139384.1 |
| AL049840.5 |
| AL513327.1 |
| GRK5-IT1 |
| AL157871.2 |
| AC092375.2 |
| LINC02804 |
| AC126773.3 |
| ITCH-IT1 |
| AC005550.1 |
| LINC01857 |
| AL731533.2 |
| AL590723.1 |
| PRDM16-DT |
| AC090617.4 |
| SNHG14 |
| AC093915.1 |
| AC025271.4 |
| AC010422.2 |
| IDI2-AS1 |
| AC087500.1 |
| LINC02435 |
| GS1-24F4.2 |
| AC104031.1 |
| AC135803.1 |
| AC025181.2 |
| AC073896.4 |
| AL445309.1 |
| LINC00989 |
| NSMCE1-DT |
| LINC01126 |
| AL109741.1 |
| LINC00336 |
| AC010883.1 |
| AP001830.1 |
| AP001469.2 |
| AC022726.1 |
| AC020907.4 |
| AC005281.1 |
| AC115618.1 |
| AC009495.1 |
| PP7080 |
| AC036108.3 |
| GLIS3-AS1 |
| LINC01983 |
| AC009318.3 |
| LINC02794 |
| AL161782.1 |
| AC012065.2 |
| AC072039.2 |
| AC079148.1 |
| AC016405.2 |
| AL356752.1 |
| AL359962.2 |
| AC040174.1 |
| AP003548.1 |
| AC137561.1 |
| ENTPD3-AS1 |
| AC096920.1 |
| AP000786.1 |
| AC067838.1 |
| AL662844.4 |
| AC105118.1 |
| AC138207.9 |
| AL136368.1 |
| AC016957.2 |
| AC103563.7 |
| AC022431.1 |
| AC009962.1 |
| AC024145.1 |
| AL139330.1 |
| PPIC-AS1 |
| AC140847.2 |
| AC011442.1 |
| AC087672.2 |
| AL590133.1 |
| AC027796.4 |
| AL023653.1 |
| DLEU1 |
| C5orf34-AS1 |
| AC079866.2 |
| AL358852.1 |
| AC022973.4 |
| AL136379.1 |
| AC108727.1 |
| AL009178.2 |
| Z98885.3 |
| AC003044.1 |
| FRY-AS1 |
| AL450384.1 |
| AC079766.1 |
| LINC01235 |
| AP001363.1 |
| C6orf223 |
| AC093585.1 |
| AC008608.2 |
| FBXO30-DT |
| AC004816.1 |
| AC097654.1 |
| AL162253.2 |
| AC113410.4 |
| AL138966.2 |
| AC093157.1 |
| AC079075.1 |
| AL133330.1 |
| LGALSL-DT |
| AC073592.1 |
| AC096733.2 |
| C22orf34 |
| AC117490.2 |
| AL136221.1 |
| AC131391.1 |
| AC007823.1 |
| LINC01521 |
| KIAA0087 |
| AL355803.1 |
| C1RL-AS1 |
| AC010359.2 |
| AC104335.1 |
| SOCAR |
| TRG-AS1 |
| AL513185.1 |
| AL139407.1 |
| AC110609.1 |
| AC010618.2 |
| Z83745.1 |
| LINC02739 |
| AC004908.2 |
| AC007996.1 |
| MINCR |
| AC011322.1 |
| AL391095.3 |
| AC139100.2 |
| AL445645.1 |
| ZBTB44-DT |
| AC093249.6 |
| FOXP4-AS1 |
| AC069257.1 |
| AC008759.2 |
| DSG2-AS1 |
| AC079907.1 |
| LINC01138 |
| HOMER3-AS1 |
| AL023803.2 |
| AP000593.3 |
| AC010201.2 |
| AC079781.5 |
| AC008663.2 |
| AL359715.3 |
| AC016727.3 |
| AL117327.1 |
| AC005082.1 |
| AC090948.2 |
| NLGN1-AS1 |
| AL139099.2 |
| AC008514.1 |
| AC080023.1 |
| AL121899.1 |
| AC097382.1 |
| LINC01802 |
| AL589935.1 |
| AC144831.1 |
| RBMS3-AS3 |
| AC015660.3 |
| AL161935.3 |
| AC011825.2 |
| AC093801.1 |
| KIF9-AS1 |
| AC138207.4 |
| HOTAIR |
| PANCR |
| AC013472.1 |
| PCCA-DT |
| KTN1-AS1 |
| AC106037.3 |
| AC010973.2 |
| AC009549.1 |
| LINC00623 |
| AC010735.2 |
| AL391987.4 |
| STXBP5-AS1 |
| AC091153.3 |
| TBX2-AS1 |
| AC100778.2 |
| TFAP2A-AS2 |
| AL023584.2 |
| AC010320.3 |
| AC012360.2 |
| AC092653.1 |
| LINC02761 |
| AL021368.3 |
| AC067930.2 |
| PSPC1-AS2 |
| AC092535.5 |
| AL161756.3 |
| ALG13-AS1 |
| SH3BP5-AS1 |
| AC078883.2 |
| AL117335.1 |
| AC022960.1 |
| HCG27 |
| MYCBP2-AS2 |
| AL583856.2 |
| SNRK-AS1 |
| AC084824.4 |
| AL606534.1 |
| RNF216-IT1 |
| AL139246.1 |
| C1QTNF1-AS1 |
| AL512408.1 |
| AC009163.7 |
| AL590096.1 |
| LINC02362 |
| AC074029.3 |
| AL136141.1 |
| NCOA7-AS1 |
| AC119396.1 |
| LINC02569 |
| LINC02076 |
| TET2-AS1 |
| AC084026.2 |
| AC087276.1 |
| AC090579.1 |
| MIR17HG |
| AC008875.3 |
| AC092279.1 |
| AC008050.1 |
| AC010245.2 |
| AL049779.4 |
| SNHG6 |
| AC004585.1 |
| MCM3AP-AS1 |
| AC005519.1 |
| AC104758.1 |
| AC000085.1 |
| LINC02427 |
| AC006213.4 |
| AP001094.3 |
| AC004832.6 |
| AL161725.1 |
| AC012645.3 |
| ZNF667-AS1 |
| LINC00865 |
| AL121772.3 |
| AC092338.3 |
| AC013403.2 |
| AC087164.1 |
| AC005498.2 |
| LINC00158 |
| ZKSCAN2-DT |
| AC016831.4 |
| AC007099.1 |
| AC025774.1 |
| AC012313.1 |
| MIR3936HG |
| LINC00571 |
| AC004233.3 |
| AC022400.1 |
| AC104333.4 |
| AC092325.1 |
| AC025176.1 |
| LMO7-AS1 |
| LINC00240 |
| AC004039.1 |
| AC005776.2 |
| AC025166.1 |
| AC108010.1 |
| AC096541.1 |
| AP001189.1 |
| AC092123.1 |
| SRRM2-AS1 |
| AC107241.1 |
| AC112220.2 |
| AL021155.4 |
| AC034102.8 |
| DGUOK-AS1 |
| AC069029.1 |
| MECOM-AS1 |
| LINC02541 |
| TMPO-AS1 |
| AC090630.1 |
| AC079768.2 |
| AC005785.1 |
| AC099568.1 |
| AL645939.4 |
| AC022007.1 |
| AC073115.2 |
| AC138305.1 |
| AC093462.1 |
| AL021154.1 |
| AP000763.3 |
| RPL37A-DT |
| AC026124.2 |
| LINC02649 |
| AL158207.2 |
| AL158167.1 |
| AL078644.1 |
| AC009119.2 |
| ADORA2A-AS1 |
| LINC02749 |
| AC083964.1 |
| AC007098.1 |
| AL031670.1 |
| AC074117.1 |
| AL589739.1 |
| AL136304.1 |
| AC026780.2 |
| AC124283.3 |
| AC096540.1 |
| AP001429.1 |
| AC112721.2 |
| AC020915.1 |
| AL121906.2 |
| AC007494.2 |
| LINC00460 |
| FGF14-IT1 |
| AL513548.4 |
| AC104984.5 |
| CKMT2-AS1 |
| AC002128.1 |
| AC011379.1 |
| AL121655.1 |
| AP000866.6 |
| AC116903.2 |
| AC025811.1 |
| RBM15-AS1 |
| AC016065.1 |
| SMIM2-IT1 |
| AL442003.1 |
| EHD4-AS1 |
| AL512506.1 |
| Z99289.2 |
| AC012467.2 |
| VIM-AS1 |
| AC012676.1 |
| AC005740.4 |
| AF186192.1 |
| MIR3142HG |
| AP000487.1 |
| AL132657.2 |
| AP001160.1 |
| LINC00663 |
| AC125618.1 |
| EGFR-AS1 |
| LINC02422 |
| AL162431.1 |
| AL121987.2 |
| AL391834.1 |
| AC008494.2 |
| AC010655.2 |
| AC007611.1 |
| AC022296.4 |
| AC008610.1 |
| AL583810.1 |
| AC010336.2 |
| AC091180.4 |
| LINC02157 |
| LINC00944 |
| AP003043.1 |
| STK24-AS1 |
| AC106818.2 |
| SSBP3-AS1 |
| AC005393.1 |
| AC063943.1 |
| AC005828.1 |
| AC244093.5 |
| AC027279.1 |
| SCARNA9 |
| ANKRD44-IT1 |
| AC010285.3 |
| AC007773.1 |
| AC110611.2 |
| AC116552.1 |
| AC114730.3 |
| AC026803.1 |
| PCCA-AS1 |
| AL118558.3 |
| AC011005.4 |
| AC002398.1 |
| AC008659.1 |
| AC010884.1 |
| FO393418.1 |
| LINC01091 |
| AC015871.3 |
| CASK-AS1 |
| AL135818.1 |
| LINC01465 |
| AP003119.2 |
| AL157402.2 |
| AC069368.2 |
| HCG18 |
| AC017048.3 |
| AC113398.2 |
| SCOC-AS1 |
| AC060766.6 |
| AL078581.4 |
| AL512770.1 |
| CFAP58-DT |
| AL139274.2 |
| LINC00686 |
| AC087878.1 |
| LINC02685 |
| LINC02062 |
| BHLHE40-AS1 |
| AC010719.1 |
| CTC-338M12.4 |
| AC034236.2 |
| AC136475.1 |
| CRYZL2P-SEC16B |
| AL139287.1 |
| AC021078.1 |
| AC139887.4 |
| AC044849.1 |
| AC053527.2 |
| RNF157-AS1 |
| AC010175.1 |
| LINC02027 |
| FENDRR |
| AC008763.1 |
| LINC02345 |
| AC000068.1 |
| PDXDC2P-NPIPB14P |
| LINC01503 |
| DCXR-DT |
| AL359546.1 |
| LINC01234 |
| IL10RB-DT |
| AL157392.3 |
| KCNIP2-AS1 |
| AC024022.1 |
| LINC02334 |
| EMC1-AS1 |
| RORA-AS1 |
| AC073046.3 |
| AC098869.2 |
| AL592295.3 |
| AL137847.1 |
| AC108058.1 |
| MELTF-AS1 |
| AC010896.1 |
| AC124947.1 |
| AL355388.1 |
| IQCH-AS1 |
| FAM153CP |
| MFF-DT |
| AP000282.1 |
| AC007406.3 |
| AL139041.1 |
| AC113143.1 |
| AC092828.1 |
| AC067945.2 |
| MIR4435-2HG |
| AC005599.1 |
| AL157904.1 |
| AC024560.4 |
| AL109936.2 |
| RASGRP3-AS1 |
| AC147067.1 |
| AC055811.3 |
| PTOV1-AS2 |
| AC016722.2 |
| AC051619.7 |
| AC092910.3 |
| AL049840.2 |
| AL157813.1 |
| AC133552.5 |
| TONSL-AS1 |
| LINC00519 |
| AC131182.1 |
| AL590652.1 |
| AL353150.1 |
| KIF1C-AS1 |
| AL355987.4 |
| AC005920.2 |
| AL049796.1 |
| AC083805.2 |
| BX323043.1 |
| AL021918.5 |
| ZMIZ1-AS1 |
| AC092111.1 |
| AL157871.5 |
| AC126118.1 |
| FAM182B |
| AC008736.1 |
| SLC6A1-AS1 |
| AC092120.3 |
| AC005253.1 |
| AL390208.1 |
| AC105020.6 |
| GNG12-AS1 |
| AC005753.1 |
| AC018766.1 |
| USP46-DT |
| ERVK13-1 |
| ITGA6-AS1 |
| AC010132.4 |
| AL139424.3 |
| AC107884.1 |
| AC008035.1 |
| STARD7-AS1 |
| AL121750.1 |
| LINC01843 |
| AP001619.1 |
| AL157392.4 |
| LINC02863 |
| AL159169.2 |
| AL356652.1 |
| PRKCQ-AS1 |
| AC104564.3 |
| AL118558.4 |
| UPP2-IT1 |
| BMS1P4 |
| AC009097.2 |
| AL132657.1 |
| RNF32-AS1 |
| AL117209.1 |
| AL451123.1 |
| AC012467.1 |
| AC016866.1 |
| AC099850.1 |
| AC020915.2 |
| RNF139-AS1 |
| AC005840.4 |
| AC010776.3 |
| RHOQ-AS1 |
| AL162430.2 |
| AL158196.1 |
| AC002128.2 |
| AC112196.1 |
| AC011379.2 |
| AC078777.1 |
| GAS6-AS1 |
| AL359922.2 |
| AC006001.2 |
| AC012653.2 |
| LINC01484 |
| MCPH1-AS1 |
| LINC01600 |
| AC092910.4 |
| AC009119.1 |
| AC127502.2 |
| AC079140.6 |
| PART1 |
| AL359220.1 |
| AC232271.1 |
| RUNX3-AS1 |
| AL139398.1 |
| VLDLR-AS1 |
| AC079313.1 |
| ZFAS1 |
| AC090772.3 |
| AC254562.3 |
| AC022382.2 |
| LINC00987 |
| RUNDC3A-AS1 |
| AP006623.1 |
| AL355075.2 |
| AC068790.2 |
| AL354733.3 |
| LINC02576 |
| KCNQ1OT1 |
| AL157786.1 |
| AL031775.1 |
| AC124014.1 |
| AP001453.2 |
| AC004884.2 |
| AC103769.1 |
| AC012306.3 |
| Z99943.1 |
| AC009560.3 |
| PDE2A-AS2 |
| AC107959.1 |
| AC131009.1 |
| GUSBP11 |
| LINC00997 |
| PSMD6-AS2 |
| FOXO6-AS1 |
| AC090517.5 |
| AL135910.1 |
| SUCLA2-AS1 |
| AC008870.4 |
| AL390955.2 |
| AP001269.4 |
| AC004554.1 |
| AC024560.3 |
| AL031432.5 |
| MADD-AS1 |
| AC012313.2 |
| AC009133.3 |
| AC108472.1 |
| AC103703.1 |
| AL135841.1 |
| AP000944.1 |
| AC091978.1 |
| PPP1R12A-AS1 |
| AC067852.5 |
| AL158151.2 |
| AC053545.1 |
| GNA14-AS1 |
| AL109946.1 |
| MIATNB |
| ELOA-AS1 |
| AL020993.1 |
| AL592546.2 |
| LINC02716 |
| DNAJC3-DT |
| AL357054.4 |
| AC004812.2 |
| COX10-AS1 |
| AC025918.1 |
| AC087071.2 |
| LINC02811 |
| AL359317.1 |
| TAPT1-AS1 |
| AC012531.1 |
| LINC01532 |
| AC011491.2 |
| LINC02606 |
| AC147067.2 |
| AC017037.5 |
| IBA57-DT |
| SMARCA5-AS1 |
| AC253536.3 |
| CCNT2-AS1 |
| AL137847.2 |
| AC006026.3 |
| AL353151.2 |
| AL353748.3 |
| AC079203.1 |
| HOXB-AS3 |
| AC008770.3 |
| AC000068.2 |
| AP000757.1 |
| AP006621.4 |
| AC010205.1 |
| AC078850.2 |
| AL731568.1 |
| NDUFB2-AS1 |
| FIGNL2-DT |
| LINC01762 |
| AC092718.4 |
| AC027682.6 |
| AC079584.2 |
| FO393419.2 |
| AC083841.1 |
| GASAL1 |
| ISM1-AS1 |
| AC131025.3 |
| OPA1-AS1 |
| ELDR |
| AC136475.2 |
| AF196972.1 |
| AC007384.1 |
| AL109924.1 |
| AL096799.1 |
| AC092164.1 |
| AC005730.3 |
| USP12-AS2 |
| AC129507.1 |
| JMJD1C-AS1 |
| LINC00630 |
| AGBL5-AS1 |
| AC003682.1 |
| LINC00885 |
| AC124854.1 |
| AL121601.1 |
| AL596442.2 |
| LINC02482 |
| AL135818.2 |
| MIR99AHG |
| AD001527.1 |
| AC009041.3 |
| AC012254.1 |
| AC139720.1 |
| LINC01123 |
| MAP3K14-AS1 |
| LINC00471 |
| AC006273.1 |
| AL731569.1 |
| AC078906.1 |
| AL645728.1 |
| DENND5B-AS1 |
| GPC6-AS2 |
| AC026803.2 |
| AC110611.1 |
| AL137127.1 |
| AC005740.3 |
| MIR223HG |
| AL450384.2 |
| FAM222A-AS1 |
| AC105105.1 |
| HOXB-AS4 |
| LINC00488 |
| LINC01699 |
| AL031289.1 |
| AC109454.3 |
| LINC02188 |
| AC097500.1 |
| AC022306.3 |
| AC090907.3 |
| AC009145.2 |
| AC096921.2 |
| AC093157.2 |
| LINC01003 |
| AATBC |
| AP001363.2 |
| AC004816.2 |
| AP000424.2 |
| CERS3-AS1 |
| FAM53B-AS1 |
| AC087222.1 |
| AC025178.1 |
| AC254633.1 |
| AC015849.1 |
| AP001767.2 |
| AC090186.1 |
| AC079174.2 |
| SLCO4A1-AS1 |
| AC006252.1 |
| LINC00894 |
| AP001107.1 |
| AL136985.1 |
| AL078604.2 |
| AC005089.1 |
| AC022146.2 |
| AC027228.1 |
| AP000919.3 |
| AC023137.1 |
| AC211476.2 |
| COL4A2-AS1 |
| AC091564.5 |
| KLHDC7B-DT |
| AC026462.3 |
| AC064801.1 |
| AL049612.1 |
| LINC01789 |
| AP002340.1 |
| LINC01094 |
| AC005014.2 |
| LINC00884 |
| CEROX1 |
| AC019205.1 |
| AC009495.2 |
| AP001554.1 |
| AL450263.1 |
| AC016405.1 |
| AC090527.3 |
| AC040174.2 |
| AC017071.1 |
| AC004947.3 |
| AL096712.2 |
| AC005696.4 |
| LINC01273 |
| AC136475.5 |
| ZNF32-AS1 |
| MATN1-AS1 |
| AL158825.2 |
| AL133367.1 |
| AL162400.2 |
| AC011510.1 |
| AC010327.6 |
| AC022726.2 |
| AC083855.2 |
| AC007849.1 |
| LINC01353 |
| AC003101.2 |
| AC006213.3 |
| AC013731.1 |
| LINC00677 |
| AL023881.1 |
| AC009464.1 |
| LINC02747 |
| AC010307.4 |
| BX255925.4 |
| MRPS30-DT |
| ZFAND2A-DT |
| AC012645.4 |
| AC087500.2 |
| AC006238.1 |
| AC004466.2 |
| AL450998.2 |
| LINC01767 |
| AC005071.1 |
| AL929236.1 |
| LINC01750 |
| LINC00528 |
| Z97192.4 |
| AC015802.5 |
| MCCC1-AS1 |
| LINC00173 |
| AC020913.3 |
| AC067852.2 |
| AC027020.2 |
| AC024270.1 |
| AC080038.1 |
| AC023906.5 |
| AC007383.1 |
| LINC01506 |
| MIR503HG |
| PICSAR |
| AC025180.1 |
| AL096828.3 |
| LINC02487 |
| AC009542.1 |
| AC008264.2 |
| AC069079.1 |
| FOXD2-AS1 |
| MCF2L-AS1 |
| PURPL |
| AL139384.2 |
| AL513327.2 |
| AC134349.1 |
| AL357874.2 |
| AC084824.3 |
| GAS6-DT |
| LINC01871 |
| AL662884.5 |
| NEAT1 |
| AC009054.1 |
| AC073896.3 |
| AL139351.1 |
| AC011523.1 |
| LINC01485 |
| SNHG7 |
| AC025271.3 |
| AP003068.2 |
| AC118344.1 |
| LINC00861 |
| AL161665.2 |
| STPG3-AS1 |
| AC241644.2 |
| AC012313.5 |
| AC087276.2 |
| NRSN2-AS1 |
| C2orf27A |
| AL022311.1 |
| AC021242.3 |
| AC109361.2 |
| AL353807.5 |
| LINC02656 |
| AL033527.3 |
| AL445205.1 |
| AC010531.3 |
| AL021707.4 |
| LINC02041 |
| SNHG8 |
| AC078883.1 |
| NFIA-AS1 |
| AC090578.2 |
| TMEM92-AS1 |
| AC021739.4 |
| SNHG30 |
| AC025154.2 |
| AL133215.2 |
| AL731566.2 |
| AC104794.3 |
| AC078909.2 |
| ZNF687-AS1 |
| AC068790.5 |
| AL359397.1 |
| AC018445.5 |
| AC138028.4 |
| AC010973.1 |
| SNHG15 |
| AC097634.1 |
| AC012360.1 |
| AL157893.2 |
| NADK2-AS1 |
| AL035461.2 |
| LINC02082 |
| AL138689.1 |
| AC245014.3 |
| AC244453.3 |
| PVT1 |
| TFAP2A-AS1 |
| LINC02544 |
| AC145285.2 |
| AC068051.1 |
| AC022272.1 |
| AC015977.1 |
| AL355102.1 |
| AL589863.1 |
| AC069549.1 |
| HOXC-AS2 |
| ZKSCAN7-AS1 |
| AL031282.2 |
| AL021392.1 |
| AC093227.1 |
| AL442663.3 |
| AC079921.2 |
| AC104316.2 |
| OCIAD1-AS1 |
| AC141002.1 |
| AC079142.1 |
| KLF7-IT1 |
| AL023803.1 |
| ARHGAP5-AS1 |
| SAP30-DT |
| LINC02285 |
| AL590764.1 |
| AC010201.1 |
| AC005332.3 |
| AC093484.4 |
| AC022137.3 |
| AC090948.1 |
| AL357514.1 |
| AC015967.1 |
| AC008663.1 |
| AL352979.4 |
| AC004817.3 |
| AC009318.4 |
| THBS3-AS1 |
| AL391840.3 |
| MAFTRR |
| GPRC5D-AS1 |
| NIPBL-DT |
| Z95118.2 |
| AC004908.1 |
| AP000813.1 |
| AC092171.4 |
| AC114321.1 |
| AC032011.1 |
| ACTA2-AS1 |
| AC099684.2 |
| AC006130.3 |
| AL353708.3 |
| AC117509.1 |
| AC008537.2 |
| AC006547.1 |
| AC018450.1 |
| MIR155HG |
| AC016394.1 |
| AC018755.4 |
| ITPRIP-AS1 |
| LINC02724 |
| AL035447.1 |
| LBX2-AS1 |
| AL662844.3 |
| AC091151.1 |
| AC211433.1 |
| AC011498.6 |
| AL021707.1 |
| AP000941.1 |
| AC006033.2 |
| SLFNL1-AS1 |
| AC083880.1 |
| LINC02185 |
| AC107375.1 |
| COL18A1-AS2 |
| AL596223.2 |
| AL121845.4 |
| AC087277.2 |
| APOA1-AS |
| AC018553.2 |
| AL357874.3 |
| HCP5 |
| AL139289.1 |
| DLGAP4-AS1 |
| AC073534.2 |
| AC018653.3 |
| AC007448.5 |
| AC011815.1 |
| AL645939.5 |
| AC067852.3 |
| AC100803.3 |
| AC118345.1 |
| AL136295.2 |
| LINC02675 |
| CDC37L1-DT |
| AC006213.2 |
| SNHG31 |
| AL139288.1 |
| LINC01507 |
| AC092428.1 |
| AC018529.2 |
| RC3H1-IT1 |
| TSPAN9-IT1 |
| FAM13A-AS1 |
| AC012435.3 |
| LINC01655 |
| AC093734.1 |
| BX537318.1 |
| AC004466.3 |
| AC009951.5 |
| AC084757.3 |
| AC010883.3 |
| AC087284.1 |
| LINC02195 |
| AC000067.1 |
| LINC00645 |
| AL133351.4 |
| MIR29B2CHG |
| AL031055.1 |
| AC008966.1 |
| VPS13B-DT |
| AL357033.2 |
| AL035661.1 |
| AP001994.2 |
| AC099778.1 |
| BX284668.5 |
| NCBP2-AS1 |
| AC244093.4 |
| AC009318.1 |
| Z82215.1 |
| AL136084.2 |
| MANEA-DT |
| AC036108.1 |
| CARNMT1-AS1 |
| MAP3K5-AS1 |
| AC011465.1 |
| AC091180.5 |
| AC025423.1 |
| AC023509.6 |
| AL031658.1 |
| AP000919.2 |
| AC022387.2 |
| PLAC4 |
| AC007663.4 |
| AC010226.1 |
| AC084117.1 |
| AC087645.2 |
| ERICH6-AS1 |
| INSYN1-AS1 |
| AC145207.8 |
| AC136424.1 |
| AC099550.1 |
| AL445228.2 |
| AC020634.2 |
| AC073655.1 |
| AL139260.1 |
| PCAT6 |
| AP001107.9 |
| FLNB-AS1 |
| AC087741.1 |
| AC016292.1 |
| AC005387.2 |
| AL355102.4 |
| AP002954.1 |
| AP001527.2 |
| EMSLR |
| AJ011932.1 |
| AL035446.1 |
| AP000345.2 |
| AC107057.1 |
| AC048382.2 |
| AP001267.3 |
| MRPL20-AS1 |
| AC105345.1 |
| AC007953.1 |
| AC079804.3 |
| AL049780.2 |
| AC011825.4 |
| C2-AS1 |
| AL137077.2 |
| PDE11A-AS1 |
| AC104984.4 |
| WWC2-AS1 |
| IRF1-AS1 |
| AC138625.1 |
| AC022364.1 |
| AL355073.2 |
| AC090907.2 |
| AC022306.2 |
| AP000525.1 |
| AL008723.2 |
| AC022217.3 |
| TMEM30A-DT |
| AC015912.3 |
| APCDD1L-DT |
| AL590560.3 |
| AC073346.1 |
| HLA-F-AS1 |
| AL133406.2 |
| AC090337.1 |
| AL109920.1 |
| BCL2L1-AS1 |
| AC034102.6 |
| MIR200CHG |
| AC108477.1 |
| AC099518.1 |
| AC005342.2 |
| AC002044.1 |
| AL035252.3 |
| AC026741.1 |
| AC112715.1 |
| ITGA9-AS1 |
| ALOX12-AS1 |
| STARD4-AS1 |
| AC092755.1 |
| AC007130.1 |
| AL512656.1 |
| PCED1B-AS1 |
| AC025034.1 |
| LHX1-DT |
| AC138150.1 |
| AC104118.1 |
| AC004817.2 |
| AL391095.1 |
| PSORS1C3 |
| AL008582.1 |
| AC087683.2 |
| AC005696.1 |
| GS1-124K5.4 |
| AC012186.2 |
| AC005332.2 |
| AC105105.4 |
| AC026356.2 |
| AC026740.1 |
| AC091849.2 |
| AC010519.1 |
| MRTFA-AS1 |
| AC107071.1 |
| AL445222.1 |
| AC016727.1 |
| AC005845.1 |
| WASHC5-AS1 |
| AC015849.4 |
| AC097382.3 |
| AC132192.2 |
| AL031709.1 |
| LINC01359 |
| CEBPB-AS1 |
| DANCR |
| AC069224.1 |
| KMT2E-AS1 |
| AP001107.4 |
| AC104232.3 |
| AL161935.1 |
| AC107993.1 |
| AC093567.1 |
| AC104564.5 |
| AC015660.1 |
| LINC01704 |
| AC022898.2 |
| MMP25-AS1 |
| AC018816.1 |
| AC008556.1 |
| AC034213.1 |
| SDCBP2-AS1 |
| AL021368.1 |
| AC024270.4 |
| AC145285.3 |
| PGM5-AS1 |
| LINC01197 |
| LCMT1-AS1 |
| MAST4-AS1 |
| LINC00667 |
| AC103740.1 |
| TXNDC12-AS1 |
| AC007066.2 |
| AC097534.1 |
| Z98884.1 |
| AC114956.1 |
| DNAJC27-AS1 |
| NARF-IT1 |
| AP000894.4 |
| AC090578.3 |
| LINC01480 |
| LINC02363 |
| AC018638.7 |
| AC027373.1 |
| AL139246.3 |
| SNHG16 |
| AL133215.3 |
| AC015922.2 |
| AC027601.1 |
| PARTICL |
| Z93930.2 |
| AL390066.1 |
| SNHG29 |
| LINC02298 |
| AL033527.2 |
| AC008875.1 |
| LIFR-AS1 |
| AC121338.2 |
| AL391121.1 |
| TMCC1-AS1 |
| LINC01614 |
| AC091488.1 |
| AP000355.1 |
| AC120498.10 |
| AC007220.1 |
| AC092338.1 |
| AC011377.1 |
| AC073896.2 |
| LINC01443 |
| AL121772.1 |
| LINC01187 |
| BX255925.1 |
| TBILA |
| BRWD1-AS2 |
| AC010422.4 |
| AC109479.1 |
| NALT1 |
| AC100830.2 |
| AC114812.2 |
| ZNF674-AS1 |
| AP001596.2 |
| AC025043.1 |
| AL354743.2 |
| LINC01705 |
| AC004233.1 |
| AC018462.1 |
| AC012073.1 |
| FOXCUT |
| LINC00339 |
| AC011752.1 |
| AC104117.3 |
| AL512652.1 |
| TNKS2-AS1 |
| LINC02577 |
| LINC01738 |
| AC105384.1 |
| AC020612.3 |
| AC018797.3 |
| AL592301.1 |
| MIRLET7A1HG |
| AC093110.1 |
| LINC01560 |
| AC092296.2 |
| LASTR |
| AL512791.1 |
| LINC01715 |
| MACORIS |
| DCUN1D2-AS |
| LINC00996 |
| AC120053.1 |
| AP000873.2 |
| HNF4A-AS1 |
| AP002490.1 |
| AC005899.5 |
| AC003984.1 |
| AC068790.3 |
| AC005632.6 |
| AC135178.6 |
| AC093797.1 |
| SCGB1B2P |
| AC104794.5 |
| LINC01251 |
| SYNE1-AS1 |
| AL160400.1 |
| AL645608.7 |
| AC109597.2 |
| AL049840.4 |
| AC092896.1 |
| AC073073.2 |
| AC034198.2 |
| AC004492.1 |
| LINC01615 |
| AC129510.1 |
| AC010531.5 |
| AC114939.1 |
| AC001226.1 |
| TEX41 |
| AL157823.2 |
| BX842570.1 |
| AC016526.2 |
| ZNF346-IT1 |
| AC010776.2 |
| AC011912.1 |
| SRGAP3-AS4 |
| AC018521.6 |
| AC022973.5 |
| AC073651.1 |
| LINC01730 |
| AC093382.1 |
| AL078581.2 |
| AC027348.1 |
| AC005332.5 |
| AC027796.5 |
| AC012443.2 |
| AC023483.1 |
| AL159169.3 |
| LINC01772 |
| AC096677.1 |
| AL157931.2 |
| AL358072.1 |
| AC090970.1 |
| LINC00662 |
| AC011461.1 |
| THCAT158 |
| AL139424.2 |
| AC098798.1 |
| AL353759.1 |
| WEE2-AS1 |
| C1QTNF7-AS1 |
| LINC02086 |
| AC137770.1 |
| AP003471.1 |
| AC023158.1 |
| AC005911.1 |
| AC063919.1 |
| LINC01182 |
| CBR3-AS1 |
| LINC01141 |
| AC009996.1 |
| AC007376.2 |
| JARID2-AS1 |
| AC023830.3 |
| AC097359.2 |
| AC073311.1 |
| AL024508.1 |
| RERG-IT1 |
| BMPR1B-DT |
| AC245884.9 |
| AC010285.1 |
| AC129507.4 |
| AC016949.1 |
| AC139887.2 |
| AL139807.1 |
| MACC1-AS1 |
| KDM4A-AS1 |
| HOXB-AS1 |
| AL096701.3 |
| TBC1D8-AS1 |
| AC138207.5 |
| AL357078.2 |
| ITPKB-IT1 |
| AC023669.2 |
| AC091181.2 |
| AL596442.3 |
| AC002563.1 |
| AC127024.6 |
| AP003721.3 |
| BICRA-AS1 |
| MAFA-AS1 |
| AL356495.1 |
| AC072061.1 |
| AC012485.2 |
| AC073323.1 |
| TP73-AS3 |
| AC008737.1 |
| ARHGAP26-IT1 |
| AC017006.2 |
| AC005479.2 |
| AC136475.3 |
| AC116351.1 |
| AL138999.1 |
| AL662797.1 |
| AL391845.2 |
| AC010501.2 |
| CA3-AS1 |
| AC107021.2 |
| AC008972.1 |
| ALDH1L1-AS2 |
| AC025031.4 |
| AC005616.1 |
| AL360182.2 |
| AC012358.2 |
| AP001781.1 |
| HHLA3 |
| AC012404.2 |
| AC008747.1 |
| ADIRF-AS1 |
| AC084398.2 |
| AC068620.1 |
| AL031848.2 |
| AC012615.6 |
| AL360157.1 |
| AL353593.2 |
| AC079015.1 |
| AC073046.1 |
| AL606834.2 |
| AL158166.2 |
| AC012640.1 |
| AL158206.1 |
| AC007406.1 |
| AC009166.1 |
| AC107959.4 |
| AL590062.1 |
| UST-AS2 |
| AC069437.1 |
| AC022165.1 |
| AC116407.2 |
| AL591002.1 |
| DDX11-AS1 |
| U62317.3 |
| TAF1A-AS1 |
| AC027801.1 |
| AC020978.7 |
| PITRM1-AS1 |
| AC084824.5 |
| CIRBP-AS1 |
| AL121672.2 |
| AP000695.1 |
| AL162171.1 |
| AC090844.2 |
| AC114284.1 |
| AL162727.2 |
| AL157838.1 |
| AC245052.4 |
| OR2A1-AS1 |
| AC092535.4 |
| SEPTIN4-AS1 |
| Z69706.1 |
| AL035587.2 |
| AC245297.2 |
| AL590282.2 |
| AC006270.1 |
| AP001628.1 |
| AL592211.1 |
| AC093752.2 |
| AL161804.1 |
| UCKL1-AS1 |
| AC022966.2 |
| OIP5-AS1 |
| AC010615.2 |
| AC003965.2 |
| AC105914.2 |
| AC007881.3 |
| AC024337.2 |
| AC010463.3 |
| EDIL3-DT |
| WWTR1-IT1 |
| AC012511.1 |
| STK32A-AS1 |
| LINC02705 |
| ZNF528-AS1 |
| AC025171.5 |
| AC245140.3 |
| AC084876.1 |
| LINC02605 |
| AC009120.3 |
| AL109659.1 |
| AC016590.2 |
| LINC01504 |
| COPDA1 |
| AC012366.1 |
| TTC28-AS1 |
| SNHG4 |
| AC005005.3 |
| SOX9-AS1 |
| AL022316.1 |
| AC137630.1 |
| AC092614.1 |
| AL121929.3 |
| AL359921.2 |
| LINC01011 |
| AC007566.1 |
| AC108673.2 |
| SSTR5-AS1 |
| AP005436.2 |
| AL078590.2 |
| CAPN10-DT |
| AL590428.1 |
| AC099795.1 |
| TFAP2E-AS1 |
| AL360270.1 |
| LINC01985 |
| AC092794.2 |
| AC092140.2 |
| AL031733.2 |
| AC000061.1 |
| AC012291.3 |
| AL137779.1 |
| MIR210HG |
| AC044840.1 |
| AL451085.1 |
| AL136115.2 |
| AC244636.3 |
| AP005131.2 |
| AC092894.1 |
| LINC01593 |
| AC006504.1 |
| AL513008.1 |
| LINC00706 |
| AC004918.1 |
| LINC02615 |
| AC106028.3 |
| ARMC2-AS1 |
| AC007292.1 |
| AC102945.1 |
| AL137244.1 |
| AC244213.1 |
| AC018904.1 |
| AL021937.3 |
| AC073257.2 |
| AP003555.2 |
| AC126121.3 |
| LINC02672 |
| AC011498.1 |
| AL110115.1 |
| AP001542.3 |
| AC092143.3 |
| AC060780.1 |
| AC093864.1 |
| AL121748.1 |
| AC002059.1 |
| AC100821.2 |
| AC005546.1 |
| LINC00886 |
| ZDHHC20-IT1 |
| BX649601.1 |
| AC112722.1 |
| AC092275.1 |
| AC005041.3 |
| AC007422.1 |
| AP002784.1 |
| AL031123.2 |
| AC093249.2 |
| AC012613.1 |
| AL161669.1 |
| AC018557.3 |
| AC009159.2 |
| AC093206.1 |
| NUTM2A-AS1 |
| AC022211.3 |
| AP000866.5 |
| AC007991.2 |
| AF127577.2 |
| AL731563.3 |
| Z97653.1 |
| AC022463.1 |
| AC135050.3 |
| AP003498.1 |
| AC087289.2 |
| AC091057.1 |
| AC005034.5 |
| AL136419.1 |
| ZBTB20-AS5 |
| AC080162.1 |
| LINC00239 |
| SHANK2-AS2 |
| AP003065.1 |
| AL365361.1 |
| AC120498.4 |
| LINC02568 |
| MAN2A1-DT |
| GSEC |
| RN7SL832P |
| AC020978.3 |
| AL133243.3 |
| Z68871.1 |
| AC138956.2 |
| RERE-AS1 |
| AC010185.1 |
| CRNDE |
| AC007879.3 |
| AC009269.5 |
| AC084357.2 |
| AF129075.3 |
| BACE1-AS |
| HM13-IT1 |
| DTNB-AS1 |
| MRPL20-DT |
| AL359853.1 |
| AC109322.1 |
| AC068790.7 |
| AGAP2-AS1 |
| AC013486.1 |
| AC025434.1 |
| AP001001.1 |
| AC011921.1 |
| LINC00863 |
| AC015727.1 |
| AC023794.4 |
| LINC02626 |
| AC008453.2 |
| AC008735.1 |
| AC104170.1 |
| AC048341.2 |
| AC103724.4 |
| AC011389.1 |
| AC026888.1 |
| AL353572.4 |
| AC104667.2 |
| AC073487.1 |
| AC079322.1 |
| FLJ37453 |
| MAGI2-AS3 |
| AP001893.3 |
| AC008937.3 |
| AC026495.1 |
| LANCL1-AS1 |
| LINC00294 |
| AL138960.1 |
| AC007497.1 |
| AC006008.1 |
| FP325332.1 |
| MIR122HG |
| AP002851.1 |
| AC012464.1 |
| AC060766.5 |
| AP002812.2 |
| AC008147.1 |
| AC093732.1 |
| CAHM |
| AC145423.2 |
| AL360169.3 |
| AL118522.1 |
| AL353803.5 |
| ARNILA |
| AC017076.1 |
| ZFHX2-AS1 |
| TRIM52-AS1 |
| NDUFA6-DT |
| AC009704.2 |
| AC007619.1 |
| AC116025.2 |
| AL356299.2 |
| AC011468.3 |
| AC027607.1 |
| AC010300.1 |
| PRKAR1B-AS1 |
| AC023813.3 |
| SEMA6A-AS1 |
| ZNF710-AS1 |
| AC008700.1 |
| AC018647.1 |
| PPM1F-AS1 |
| AC027271.1 |
| AC096708.3 |
| AC002480.1 |
| AC091982.1 |
| THAP9-AS1 |
| SNHG25 |
| AL732292.2 |
| AC004461.2 |
| ERI3-IT1 |
| AC100810.3 |
| ITGB2-AS1 |
| AC002064.2 |
| AC027449.1 |
| AC024075.1 |
| AL136988.2 |
| FSIP2-AS1 |
| AC145207.5 |
| AP001972.4 |
| AC007485.1 |
| DIAPH1-AS1 |
| AC006160.1 |
| AC011462.4 |
| AL353804.2 |
| AC092953.2 |
| AL022238.2 |
| AC058791.1 |
| AC125494.2 |
| AP001610.2 |
| AC025175.2 |
| AC093673.1 |
| AL136531.1 |
| AC246817.1 |
| AP001178.1 |
| RCCD1-AS1 |
| AC005775.1 |
| AL391261.2 |
| AC005865.2 |
| AC099482.1 |
| AC007684.2 |
| LNX1-AS2 |
| AC124017.1 |
| AC009148.1 |
| AC008667.1 |
| AC007405.4 |
| RAD51-AS1 |
| AC026368.1 |
| AC005224.3 |
| LINC00092 |
| GEMIN7-AS1 |
| ASB16-AS1 |
| AC136469.1 |
| MIR34AHG |
| AC130371.2 |
| AC009754.1 |
| AL078459.1 |
| AC121247.1 |
| NFE2L1-DT |
| AL022341.1 |
| MAPKAPK5-AS1 |
| AL359643.2 |
| AL596325.2 |
| ARHGAP26-AS1 |
| AC008250.2 |
| AC106873.1 |
| AL353801.3 |
| AC005154.4 |
| DNM1P35 |
| AC002310.1 |
| LINC01841 |
| AC025165.5 |
| AL031667.3 |
| AC245884.10 |
| AC117500.2 |
| AC010809.1 |
| AL049840.6 |
| EPB41L4A-AS1 |
| AC127540.2 |
| AC135178.4 |
| AC087362.2 |
| TNS1-AS1 |
| AC073326.1 |
| AL355432.1 |
| AC084781.1 |
| PKP4-AS1 |
| Z69666.1 |
| AC079380.1 |
| AC104534.1 |
| PRR34 |
| AL080317.1 |
| AC132872.3 |
| AC035139.1 |
| AC023632.2 |
| AC084024.4 |
| AC087286.1 |
| AC018695.9 |
| AL117336.2 |
| Z94721.3 |
| AF064858.2 |
| AC010168.1 |
| LINC01023 |
| AC007390.1 |
| AL451069.1 |
| AL365272.1 |
| AC011726.2 |
| EIF3J-DT |
| AL137785.1 |
| AC092127.2 |
| RAB11B-AS1 |
| AC067750.1 |
| BET1-AS1 |
| AL513165.1 |
| AL023882.1 |
| AC093824.2 |
| AL591721.1 |
| AL136038.4 |
| AC063960.1 |
| AC007728.3 |
| AL590064.1 |
| HPN-AS1 |
| LINC01612 |
| AC008569.2 |
| AC005746.3 |
| AC095057.3 |
| CXXC5-AS1 |
| AC093001.1 |
| AL353803.2 |
| AC100861.1 |
| AL078587.2 |
| AC091132.2 |
| AP002812.5 |
| AC002553.1 |
| OVCH1-AS1 |
| AC011477.1 |
| DLEU2L |
| LINC01311 |
| AL928921.1 |
| AC092542.1 |
| AC005062.1 |
| AL133476.1 |
| AC012378.2 |
| GATA6-AS1 |
| AC004863.1 |
| DSCAM-AS1 |
| AC009090.3 |
| TNFRSF10A-AS1 |
| AL513218.1 |
| AL031963.3 |
| AC103739.3 |
| MIRLET7BHG |
| AC006111.3 |
| AC020658.5 |
| STARD13-AS |
| AL355304.1 |
| AL353622.2 |
| AC004263.2 |
| AL132780.1 |
| DIRC3 |
| AC137932.1 |
| ODF2-AS1 |
| AC087392.4 |
| AL928654.2 |
| AL158163.2 |
| AC012368.2 |
| AC107081.3 |
| AP003071.1 |
| AL031320.2 |
| C9orf139 |
| AC010642.2 |
| AC127024.4 |
| LINC00909 |
| AC005034.2 |
| AL031768.2 |
| AC087289.5 |
| AC004846.2 |
| AC008731.1 |
| AL359878.2 |
| AC012085.2 |
| AC018761.1 |
| AC022079.2 |
| AL138921.2 |
| AL354836.1 |
| HMGN3-AS1 |
| AC127070.2 |
| MAMDC2-AS1 |
| AC009269.2 |
| AP000766.1 |
| AC068205.2 |
| AC110995.1 |
| AC037459.4 |
| AL355377.3 |
| AC090018.2 |
| AC022558.1 |
| AC010331.1 |
| AC005410.2 |
| AL008729.1 |
| AC008434.1 |
| AL356740.1 |
| AC015819.1 |
| LINC02884 |
| AC027097.2 |
| AC002091.1 |
| AC087752.3 |
| AC090912.2 |
| AC009268.2 |
| LINC01768 |
| EIPR1-IT1 |
| AC004494.1 |
| PABPC4-AS1 |
| AL158834.2 |
| AC023494.1 |
| AC243960.3 |
| AL390728.4 |
| AC046143.1 |
| AC112496.1 |
| AC010761.3 |
| AL589745.1 |
| AC010624.5 |
| LINC02803 |
| AC010999.1 |
| AC105020.1 |
| AC016026.1 |
| LINC01290 |
| AC012076.1 |
| AL133255.1 |
| AL031123.5 |
| AF111169.3 |
| AC068733.3 |
| AC092574.2 |
| AUXG01000058.1 |
| LINC-PINT |
| WDFY3-AS2 |
| AL034417.3 |
| CDC42-IT1 |
| AC008554.1 |
| AL139349.1 |
| AC006064.1 |
| AC231981.1 |
| AC069234.1 |
| AC092944.1 |
| AP4B1-AS1 |
| AL592431.1 |
| CCDC28A-AS1 |
| AC006480.2 |
| AC007877.1 |
| AL132989.1 |
| LINC02390 |
| AC004870.4 |
| AC007601.2 |
| AC021205.3 |
| AC091435.2 |
| AC090197.1 |
| AL356481.1 |
| AL022328.3 |
| AC023421.1 |
| AL158071.2 |
| AC016737.1 |
| AC012615.4 |
| AC079922.2 |
| AC004832.5 |
| AC019197.1 |
| AL050327.1 |
| SAPCD1-AS1 |
| LINC02019 |
| AC061975.8 |
| LINC02604 |
| AL121935.1 |
| AL356218.2 |
| AP005131.5 |
| AC018695.4 |
| LINC02343 |
| AC119800.1 |
| AC104841.1 |
| AC120349.1 |
| SNHG1 |
| AL929091.1 |
| AC004000.1 |
| AC003070.1 |
| AC108134.2 |
| AC011365.1 |
| AC099811.5 |
| AC112484.1 |
| LINC02528 |
| AC005532.1 |
| AC012409.1 |
| LINC02640 |
| AC092376.1 |
| LINC01515 |
| BACH1-IT1 |
| AC010273.2 |
| AC005261.1 |
| NNT-AS1 |
| PLCB1-IT1 |
| SUGT1-DT |
| PIK3CD-AS2 |
| LINC02614 |
| AC114760.2 |
| AC078846.1 |
| SBF2-AS1 |
| LINC02693 |
| AL512603.2 |
| LINC00487 |
| AC025171.2 |
| AC017116.2 |
| AC089999.2 |
| LINC02449 |
| ZNF337-AS1 |
| AP001625.2 |
| AL359762.3 |
| AL390195.2 |
| Z94721.1 |
| AP003352.1 |
| AL161909.2 |
| AC116158.3 |
| SKAP1-AS1 |
| AC106870.1 |
| AC005291.2 |
| AC012557.2 |
| LINC01909 |
| AC006445.2 |
| AC109460.2 |
| AC092329.4 |
| AC069200.1 |
| AC006058.3 |
| ATP1A1-AS1 |
| AC104964.3 |
| AC103739.2 |
| AL031963.2 |
| AC074138.1 |
| LINC02084 |
| AC005899.7 |
| AC092756.1 |
| AC004069.1 |
| AL596087.2 |
| AC006111.2 |
| AC073912.2 |
| ZNF350-AS1 |
| AC005746.2 |
| AC008764.8 |
| AC087588.1 |
| AC025430.1 |
| LINC01678 |
| AC005837.4 |
| AP005900.1 |
| TRIM7-AS1 |
| ITPR1-DT |
| AC079848.1 |
| AC124067.2 |
| AC073593.2 |
| AC108860.2 |
| AC016027.1 |
| AP001372.2 |
| FAM66D |
| TMEM202-AS1 |
| MICB-DT |
| AC006435.2 |
| AC127526.5 |
| AL158835.2 |
| RELA-DT |
| PELATON |
| AC087301.1 |
| AC103706.1 |
| POT1-AS1 |
| AC021683.1 |
| AC007728.2 |
| U73169.1 |
| P4HA2-AS1 |
| AC015871.6 |
| LINC02273 |
| AC004837.2 |
| COLCA1 |
| AC004034.1 |
| AC012213.3 |
| AC023825.2 |
| AC103591.3 |
| AC132872.2 |
| AC087742.1 |
| AL354760.1 |
| AL606491.1 |
| AP006545.1 |
| LINC01431 |
| AL157700.1 |
| SPIN4-AS1 |
| AC018809.2 |
| AC004825.2 |
| AC244035.1 |
| NINJ2-AS1 |
| AC010969.2 |
| AC092802.1 |
| AL590822.1 |
| AL022322.1 |
| AP001029.2 |
| AL355001.1 |
| YEATS2-AS1 |
| AC131097.2 |
| AC008555.1 |
| AC092078.3 |
| AC026471.2 |
| AL358075.1 |
| AC011468.5 |
| UBE2D3-AS1 |
| MIAT |
| AC103810.2 |
| AL607028.1 |
| AC004771.2 |
| AC015853.1 |
| LINC01473 |
| AC005180.2 |
| LINC01397 |
| C20orf197 |
| AL109615.4 |
| AC069234.4 |
| PPP3CB-AS1 |
| AL162274.1 |
| SPON1-AS1 |
| AC015911.3 |
| FGF12-AS3 |
| AC078788.1 |
| AL121944.1 |
| KRT7-AS |
| AF131215.4 |
| AC130469.1 |
| CPNE8-AS1 |
| LINC02453 |
| AL353801.2 |
| RSF1-IT2 |
| GK-IT1 |
| AC079210.1 |
| AL031727.2 |
| AC007285.1 |
| AC006042.3 |
| RERG-AS1 |
| AL121917.1 |
| HCFC1-AS1 |
| AC009961.3 |
| AP000350.6 |
| AC005332.7 |
| ZNF436-AS1 |
| AC007785.1 |
| AC026470.2 |
| PAXBP1-AS1 |
| AL031666.3 |
| AC138393.2 |
| SEPSECS-AS1 |
| AC087749.2 |
| SRP14-AS1 |
| AP001381.1 |
| AC022154.1 |
| AP000911.1 |
| AL590729.1 |
| AC025171.3 |
| AC068722.2 |
| ZRANB2-AS2 |
| AC010247.1 |
| AC130324.1 |
| AC092809.2 |
| AL121890.4 |
| AC090515.2 |
| AC087286.4 |
| LINC00939 |
| PRR7-AS1 |
| AC092652.2 |
| AC110285.2 |
| AC010319.3 |
| AC104472.4 |
| AC108134.3 |
| AP001636.3 |
| AC010333.1 |
| AC024451.4 |
| AL022328.2 |
| AC020661.1 |
| AC025754.2 |
| RTCA-AS1 |
| AC007347.1 |
| SUGCT-AS1 |
| CTBP1-DT |
| AC007743.1 |
| AC138207.7 |
| AL157395.1 |
| AC005586.1 |
| HIF1A-AS3 |
| AC007598.1 |
| AC092168.2 |
| NORAD |
| LINC00892 |
| AL355999.1 |
| BTG3-AS1 |
| AP000851.2 |
| AC093675.1 |
| AC104819.3 |
| AC017104.5 |
| AC073842.2 |
| SEMA3F-AS1 |
| AC023510.2 |
| AL136295.7 |
| VCAN-AS1 |
| LYRM4-AS1 |
| AC010271.2 |
| Z98884.2 |
| AC099521.3 |
| LINC01535 |
| AL034417.2 |
| LIX1L-AS1 |
| RNF213-AS1 |
| AL355916.2 |
| UICLM |
| AC010261.2 |
| CASC2 |
| AL356361.2 |
| AC022916.1 |
| AC009163.4 |
| SDK1-AS1 |
| AL161630.1 |
| AL137186.1 |
| PTOV1-AS1 |
| AC027682.4 |
| AC107027.3 |
| AC011466.1 |
| FAF1-AS1 |
| AL135999.3 |
| CTBP1-AS |
| AL591848.1 |
| HCG15 |
| LAMC1-AS1 |
| AC040977.1 |
| AC010487.1 |
| AC084036.1 |
| FAM245A |
| AP000692.1 |
| LINC00702 |
| ATXN2-AS |
| HSD11B1-AS1 |
| AL358472.4 |
| MIR31HG |
| AL035416.1 |
| AC083837.1 |
| MDS2 |
| AL445490.1 |
| TYMSOS |
| AC138932.4 |
| AC016396.1 |
| AC135050.5 |
| AC036214.2 |
| AC006213.7 |
| NAV2-AS3 |
| AC124242.1 |
| PXN-AS1 |
| HOXA10-AS |
| ATP6V0E2-AS1 |
| AC011899.2 |
| STX18-AS1 |
| SEPTIN9-DT |
| LINC01534 |
| AC245060.6 |
| AL359095.1 |
| AC112503.2 |
| AC020978.2 |
| AC008734.1 |
| AC096586.2 |
| AC005153.1 |
| AC243964.3 |
| AC010542.5 |
| Z84485.1 |
| AL353796.1 |
| AL133243.2 |
| AC092720.3 |
| AC026333.4 |
| AC010976.1 |
| AC015726.1 |
| AC007342.4 |
| LINC02608 |
| AC010904.2 |
| AC009052.1 |
| AL731563.2 |
| AC022509.3 |
| AC008982.2 |
| AC114811.2 |
| AC012181.2 |
| AL109761.1 |
| AC106786.1 |
| AC093904.2 |
| AL645940.1 |
| AC019080.1 |
| LINC01213 |
| AC087623.2 |
| AC018557.2 |
| AC007278.1 |
| DNM3OS |
| AC008014.1 |
| AC026979.3 |
| AC011939.2 |
| AC009159.3 |
| AC007216.4 |
| AC099518.6 |
| ZSCAN16-AS1 |
| AC024896.1 |
| AC023794.1 |
| AL627171.2 |
| AC008115.1 |
| AC007496.1 |
| AC008735.4 |
| LINC02021 |
| AC020917.2 |
| AL034374.1 |
| LAMA5-AS1 |
| AL391117.1 |
| LOH12CR2 |
| AC017067.1 |
| AC008781.1 |
| AP005131.3 |
| AC233280.1 |
| LINCR-0001 |
| AC008551.1 |
| AC068279.2 |
| AC090559.2 |
| AC018690.1 |
| GRASLND |
| SP2-AS1 |
| RPARP-AS1 |
| AC106028.2 |
| Z97055.2 |
| CCDC18-AS1 |
| AC104653.2 |
| AL353699.1 |
| COA6-AS1 |
| LINC00921 |
| AC124944.3 |
| AC116667.2 |
| SEC24B-AS1 |
| AP001178.4 |
| AC108673.3 |
| AC079209.2 |
| KANSL1L-AS1 |
| AC010536.2 |
| AC004076.2 |
| AC011472.1 |
| TMCO1-AS1 |
| AC018752.1 |
| LINC01943 |
| Z98200.1 |
| AL031600.1 |
| AC007993.2 |
| AC103746.1 |
| AC144548.1 |
| AC009120.2 |
| AC008957.1 |
| AC004951.4 |
| AC245140.2 |
| SLC2A1-AS1 |
| AC124016.1 |
| UGDH-AS1 |
| AC113139.1 |
| CFLAR-AS1 |
| AL133370.1 |
| AC079035.1 |
| Z82185.1 |
| AL121929.2 |
| AC112907.3 |
| AC078922.1 |
| AC108053.1 |
| AC005264.1 |
| AC008124.1 |
| AC099811.3 |
| AC010997.2 |
| LINC01545 |
| AL162724.2 |
| AC067817.1 |
| LINC02303 |
| AC009554.2 |
| AC243829.2 |
| AL391001.1 |
| AP000640.1 |
| AC026992.2 |
| AC090515.5 |
| AL451050.2 |
| AC010463.2 |
| MUC20-OT1 |
| AC068234.2 |
| AC025580.3 |
| AP005482.2 |
| AC103809.1 |
| AC011416.3 |
| AL034550.3 |
| AC040169.1 |
| AC011444.2 |
| SMAD9-IT1 |
| AC079384.1 |
| EXOC3-AS1 |
| AC092053.4 |
| AC004112.1 |
| HCG25 |
| LINC01522 |
| UBAC2-AS1 |
| AL442125.2 |
| AC104530.1 |
| AC188616.1 |
| ARHGAP27P1-BPTFP1-KPNA2P3 |
| AL133297.2 |
| AC145098.1 |
| PRRT3-AS1 |
| KDM7A-DT |
| SNHG19 |
| AL158212.1 |
| AC011503.1 |
| MIR1915HG |
| AL022344.1 |
| AC009974.2 |
| TCL6 |
| REV3L-IT1 |
| AC009812.1 |
| AP000553.2 |
| NCK1-DT |
| HOXB-AS2 |
| AC008543.3 |
| AC011479.2 |
| AC006960.2 |
| AC103702.1 |
| AC005670.3 |
| AL035467.2 |
| LINC01606 |
| AF127936.1 |
| ZNF213-AS1 |
| AC008443.4 |
| AC096708.2 |
| LINC02532 |
| AC009309.1 |
| MALINC1 |
| AC104596.1 |
| LINC00628 |
| NKILA |
| AL360181.2 |
| LINC00540 |
| AC110792.3 |
| AC132872.5 |
| AC090510.2 |
| LINC00271 |
| AP001458.1 |
| LINC01801 |
| SNHG26 |
| AL138963.1 |
| AC103810.5 |
| LINC01550 |
| AL021707.6 |
| AC092902.6 |
| AC092295.2 |
| ETV7-AS1 |
| AC114316.1 |
| AL162586.1 |
| TM4SF19-AS1 |
| BISPR |
| AL035071.2 |
| AP001330.4 |
| AC004381.1 |
| AP002812.3 |
| AP000255.1 |
| AC093424.1 |
| LINC02762 |
| AC134775.1 |
| AP005264.1 |
| LINC00323 |
| AL121820.1 |
| AL161668.4 |
| RPS6KA2-IT1 |
| AC006272.1 |
| AP001793.1 |
| AL162377.1 |
| AC130650.1 |
| LNCSRLR |
| F10-AS1 |
| AC007622.2 |
| AC004223.4 |
| AC008937.2 |
| AC034234.1 |
| LINC01886 |
| AC022537.1 |
| AC116348.1 |
| AC013549.1 |
| AC136604.2 |
| AC096992.2 |
| AC032044.1 |
| AC011481.2 |
| AC084740.1 |
| LINC01239 |
| AC110769.2 |
| PTPRJ-AS1 |
| AC025370.2 |
| Z97200.1 |
| AC020558.2 |
| AL592148.3 |
| ARHGAP31-AS1 |
| AC016493.1 |
| AC118553.1 |
| AC019257.2 |
| LINC01896 |
| AC113361.1 |
| UBE2Q1-AS1 |
| AC099786.1 |
| AP001432.1 |
| AC104971.2 |
| FGF14-AS1 |
| AC021188.1 |
| PCA3 |
| LINC01637 |
| GATA3-AS1 |
| AL161669.4 |
| AP000867.5 |
| SLC16A1-AS1 |
| AC067817.2 |
| AL354707.1 |
| C21orf62-AS1 |
| LINC00551 |
| MIR194-2HG |
| AL137002.2 |
| AC105137.2 |
| AP002360.1 |
| AC005899.6 |
| LINC01270 |
| AP000696.2 |
| AC005104.1 |
| TSPOAP1-AS1 |
| AL162724.1 |
| AL031705.1 |
| AL356419.1 |
| AC243829.1 |
| RBM26-AS1 |
| AC025048.4 |
| AC026992.1 |
| AL109809.4 |
| AC008467.1 |
| ACBD3-AS1 |
| C9orf147 |
| AC068768.1 |
| AL356124.2 |
| AC091114.1 |
| AC245884.8 |
| AC010531.6 |
| AC104779.1 |
| AC025164.1 |
| AL024498.1 |
| AC008957.2 |
| AL512306.2 |
| MAILR |
| AC009087.1 |
| NOP53-AS1 |
| AC100823.1 |
| LINC01489 |
| LINC02561 |
| OTUD6B-AS1 |
| TRHDE-AS1 |
| AC004943.1 |
| AC025766.1 |
| LINC01232 |
| NR2F1-AS1 |
| HS1BP3-IT1 |
| AC106782.5 |
| AC016252.1 |
| AC092301.1 |
| AC016355.1 |
| AC010536.1 |
| AC068338.3 |
| AC073611.1 |
| AC116667.1 |
| AL512283.2 |
| AC004765.1 |
| AC025165.4 |
| LINC02669 |
| AL031186.1 |
| AC002542.6 |
| AC244153.1 |
| AC105429.1 |
| PTPRK-AS1 |
| AC011472.2 |
| AC012236.1 |
| AL033397.2 |
| SNED1-AS1 |
| AC107294.1 |
| AC004918.3 |
| AC090559.1 |
| AC005383.1 |
| FLJ20021 |
| AL358780.1 |
| LINC00659 |
| AC008781.2 |
| AC123768.1 |
| AL133371.2 |
| SPAG5-AS1 |
| AC104653.1 |
| AC008543.4 |
| AP004609.3 |
| AC005520.5 |
| AC007292.3 |
| AC022706.1 |
| AL513320.1 |
| AC244197.2 |
| ST3GAL6-AS1 |
| LINC01389 |
| AL139123.1 |
| AL157932.1 |
| AC007496.2 |
| AL161729.3 |
| LINC02432 |
| AL807757.2 |
| LINC01322 |
| LINC02289 |
| AC126175.2 |
| ZNF32-AS2 |
| AP002907.1 |
| AC023794.2 |
| AC097532.3 |
| AC007878.1 |
| LENG8-AS1 |
| AC022210.1 |
| LINC01018 |
| LINC01160 |
| AC090198.1 |
| LINC02773 |
| LINC01278 |
| AC092807.3 |
| AC090607.1 |
| AC064875.1 |
| AL670729.3 |
| AL589669.1 |
| AL132655.2 |
| MIR4453HG |
| NFE4 |
| CD44-AS1 |
| AC090825.1 |
| AL161669.3 |
| AC017104.1 |
| AC010524.1 |
| AL139243.1 |
| AL117339.3 |
| AC096642.1 |
| AC093525.4 |
| AC022211.1 |
| AC018521.5 |
| AL355488.1 |
| AC024361.3 |
| AP001271.1 |
| AL358115.1 |
| AC008496.2 |
| PLCG1-AS1 |
| AC012181.1 |
| XXYLT1-AS2 |
| AC020978.1 |
| AC005096.1 |
| AC087318.1 |
| AP003032.1 |
| AC006441.1 |
| PTPRN2-AS1 |
| AC074124.1 |
| AC010976.2 |
| AF129075.1 |
| MANCR |
| AP003390.1 |
| AL133243.1 |
| SEMA3B-AS1 |
| GSN-AS1 |
| LUCAT1 |
| AP000942.5 |
| AL121832.3 |
| AL121933.2 |
| AC007216.3 |
| SUCLG2-AS1 |
| AC026979.4 |
| AL031123.4 |
| AL157911.1 |
| AL139021.1 |
| AC007365.1 |
| AC027763.2 |
| AC073575.2 |
| AL121821.2 |
| LINC02356 |
| AC097639.1 |
| GLYCTK-AS1 |
| LINC00862 |
| AC011481.1 |
| FARSA-AS1 |
| AC005759.1 |
| AL645608.1 |
| ST7-AS1 |
| AC108471.3 |
| SEC62-AS1 |
| MRPS9-AS1 |
| AC092839.1 |
| LINC01376 |
| AC120498.2 |
| LINC00906 |
| AP001893.1 |
| ANKRD33B-AS1 |
| GABPB1-AS1 |
| AL354953.1 |
| AC130650.2 |
| ATP1B3-AS1 |
| HLX-AS1 |
| AL590226.1 |
| AC027702.1 |
| MMEL1-AS1 |
| AC007272.1 |
| PACERR |
| SAP30L-AS1 |
| LINC01928 |
| AL513477.2 |
| AC015961.2 |
| RAB30-DT |
| AL603832.1 |
| AC108693.2 |
| AC007991.4 |
| AF127577.4 |
| AC130352.1 |
| AP003120.1 |
| AL121820.2 |
| AL354928.1 |
| AL590666.1 |
| AC087045.2 |
| AC127024.5 |
| USP30-AS1 |
| DNAJB5-DT |
| SLC12A9-AS1 |
| CLMAT3 |
| AC068533.3 |
| AL031058.1 |
| AL513550.1 |
| AC137834.2 |
| AC016590.4 |
| AC011468.1 |
| CARMN |
| AC118755.1 |
| AL137009.1 |
| AL035071.1 |
| AC073367.1 |
| AC009315.1 |
| AC108463.2 |
| AL162586.2 |
| U91328.3 |
| SNHG5 |
| LINC02006 |
| AC108488.3 |
| AC084048.1 |
| CCDC144NL-AS1 |
| AC068989.1 |
| YTHDF3-AS1 |
| AL392048.1 |
| AC099811.4 |
| AC103702.2 |
| AC002347.2 |
| AC009032.1 |
| AC009113.1 |
| AL050343.2 |
| U52111.1 |
| AC091946.2 |
| AC079305.2 |
| AC022167.3 |
| AL360181.1 |
| AC073263.1 |
| AC009974.1 |
| AC015914.1 |
| AP005131.4 |
| AP001062.1 |
| C6orf99 |
| TMEM254-AS1 |
| GAS8-AS1 |
| AC004832.4 |
| AP000915.2 |
| AC124319.1 |
| AL442125.1 |
| AC009336.1 |
| AC011444.1 |
| AP001178.3 |
| AL513190.1 |
| AF230666.1 |
| CH17-340M24.3 |
| AC099552.5 |
| AC116651.1 |
| AC104365.2 |
| MYOSLID |
| AC156455.1 |
| AL158212.2 |
| AL133297.1 |
| CARD8-AS1 |
| AC009951.6 |
| AC012447.1 |
| AL357075.2 |
| AC007681.1 |
| AC021851.1 |
| AC100763.1 |
| AL158071.4 |
| AC007938.3 |
| UBR5-AS1 |
| AC073636.1 |
| AP001053.1 |
| AC005674.1 |
| AL121917.2 |
| AC011306.1 |
| AL162274.2 |
| LINC00511 |
| AC090409.1 |
| AC005180.1 |
| AL158212.5 |
| AL138995.1 |
| AC005329.3 |
| AC245060.7 |
| AC004870.2 |
| CHROMR |
| AP000240.1 |
| AC078788.2 |
| AC078962.1 |
| AC002070.1 |
| AC253536.6 |
| AC107214.1 |
| CACTIN-AS1 |
| AL353801.1 |
| AC026471.1 |
| AP001029.1 |
| AC092802.2 |
| AL355001.2 |
| AC008555.2 |
| AL157394.3 |
| AL121782.1 |
| AC093510.1 |
| AC025171.4 |
| AC004771.1 |
| AC103691.1 |
| AC009404.1 |
| AL049552.1 |
| ZNF775-AS1 |
| AC004449.1 |
| RGMB-AS1 |
| AP006545.2 |
| AC092162.2 |
| Z97832.2 |
| LINC00426 |
| AC090589.3 |
| AC026369.3 |
| AC132872.1 |
| AC022126.1 |
| AP001107.8 |
| SERPINB9P1 |
| AC002550.2 |
| MIR3659HG |
| LINC01814 |
| AL445524.1 |
| AC018809.1 |
| MGAT3-AS1 |
| AC010319.4 |
| AC022613.3 |
| NPTN-IT1 |
| LINC01410 |
| LINC01089 |
| AC233728.1 |
| ADAMTSL4-AS2 |
| LINC02154 |
| AP001372.1 |
| TSPEAR-AS1 |
| AC021683.2 |
| AC011443.1 |
| AL358472.3 |
| SENCR |
| AC133550.3 |
| AL031716.1 |
| FMR1-IT1 |
| AC090004.2 |
| AC018648.1 |
| AL157834.4 |
| AC005746.1 |
| AL355312.2 |
| AC127024.2 |
| HECW2-AS1 |
| ZBTB20-AS4 |
| AGAP1-IT1 |
| AC107464.2 |
| AC016027.2 |
| AC011477.3 |
| AC053513.2 |
| AP002518.2 |
| AL049539.1 |
| AC005790.1 |
| WAKMAR2 |
| AC009090.1 |
| MUC12-AS1 |
| LINC01703 |
| AC009716.1 |
| AC009902.2 |
| AL008730.1 |
| LINC01117 |
| AC109460.1 |
| AL356094.2 |
| AC012593.2 |
| AC073912.1 |
| LINC02585 |
| AC103739.1 |
| AC004847.1 |
| AC022144.1 |
| MGC27382 |
| AC068580.3 |
| FLVCR1-DT |
| EP300-AS1 |
| AC105219.1 |
| USP2-AS1 |
| AL390195.1 |
| CDK6-AS1 |
| AP003419.3 |
| AC108449.2 |
| AC017099.2 |
| AC107081.1 |
| AC012557.1 |
| AC137932.3 |
| EIF1B-AS1 |
| LINC02521 |
| AL139124.1 |
| AL158063.1 |
| AP000346.1 |
| AC073316.2 |
| AL603756.1 |
| AC068492.1 |
| AC099542.1 |
| AC044781.1 |
| LINC01237 |
| AC100793.3 |
| AC016586.1 |
| RAD21-AS1 |
| AC091212.1 |
| AL049871.1 |
| AL096865.1 |
| AC018761.3 |
| AC010260.1 |
| AC027514.2 |
| INTS6-AS1 |
| IGBP1-AS1 |
| AL359915.1 |
| AL139280.1 |
| AC011451.3 |
| AL591848.2 |
| AC114763.1 |
| AL356740.3 |
| AP002993.1 |
| LINC02115 |
| AC022558.3 |
| AC020663.3 |
| LINC02812 |
| AP000692.2 |
| AC074132.1 |
| EML4-AS1 |
| LINC02416 |
| ALMS1-IT1 |
| AC010261.1 |
| AL132800.1 |
| AC055855.1 |
| AC010761.1 |
| MAP3K2-DT |
| AC243960.1 |
| AL137186.2 |
| AC034102.5 |
| AL512363.1 |
| AL021707.7 |
| AL020997.3 |
| AL133551.1 |
| AC023510.1 |
| ITFG1-AS1 |
| AC024909.1 |
| JPX |
| OBSCN-AS1 |
| LINC02802 |
| AC010271.1 |
| C15orf56 |
| AP001033.2 |
| FUT8-AS1 |
| AL109976.1 |
| BAIAP2-DT |
| AC009283.1 |
| AC092747.4 |
| AC007598.2 |
| ZBED3-AS1 |
| AL109615.3 |
| UBL7-AS1 |
| AC006064.3 |
| AL356481.3 |
| IGFL2-AS1 |
| AL591895.1 |
| AC008750.4 |
| SNHG22 |
| AFAP1-AS1 |
| LINC02437 |
| AC009093.4 |
| AC083870.1 |
| AC010333.2 |
| AC017083.1 |
| AL022328.1 |
| AC064836.3 |
| KDM2B-DT |
| AC087239.1 |
| AC037198.1 |
| AC005856.1 |
| AC011462.5 |
| USP12-AS1 |
| POLR2J4 |
| AC024267.5 |
| LNCTAM34A |
| LINC01569 |
| LINC02481 |
| ZRANB2-AS1 |
| AC099343.2 |
| AC103591.4 |
| AC010247.2 |
| AL359504.1 |
| AC004257.1 |
| ETV5-AS1 |
| UBA6-AS1 |
| AL390719.3 |
| AC012409.3 |
| AC112484.3 |
| LINC00472 |
| TSBP1-AS1 |
| LINC00924 |
| PRANCR |
| LINC02875 |
| AC135178.2 |
| LINC02391 |
| AC087749.1 |
| AL590617.2 |
| AC021087.1 |
| AC103858.1 |
| AC072022.1 |
| AC016747.3 |
| AL022724.3 |
| AC093788.1 |
| AC092376.3 |
| LIMS1-AS1 |
| KLRK1-AS1 |
| AC034139.1 |
| AC000120.1 |
| TCEAL3-AS1 |
| LINC00845 |
| AC002451.1 |
| AC084125.2 |
| AC037487.2 |
| AC131953.1 |
| LINC01106 |
| AL033381.3 |
| LINC01220 |
| FTX |
| AC004263.1 |
| AL353622.1 |
| AL133227.1 |
| HLA-DQB1-AS1 |
| AL158163.1 |
| AC012368.1 |
| AL157762.1 |
| AC007569.1 |
| B4GALT1-AS1 |
| AC137932.2 |
| AC104938.1 |
| AC116158.1 |
| AC005695.3 |
| AL022162.1 |
| AC015987.1 |
| LINC01415 |
| CHRM3-AS1 |
| AC068987.2 |
| AC040904.1 |
| Z97353.2 |
| AC091965.1 |
| AL034417.4 |
| AC078856.1 |
| AP001207.3 |
| AC092954.1 |
| AC034229.4 |
| AL353803.1 |
| KIAA1671-AS1 |
| AL161430.1 |
| RHOA-IT1 |
| LINC02012 |
| DM1-AS |
| AC006059.1 |
| AC127024.3 |
| AC011477.2 |
| AC016027.3 |
| AC136601.1 |
| AL021707.8 |
| HCG14 |
| AC002553.2 |
| LINC02328 |
| AC134407.1 |
| AL731702.1 |
| EPS15-AS1 |
| LINC01252 |
| AL359541.1 |
| AC012150.2 |
| AC007342.5 |
| AP000907.2 |
| AC011676.1 |
| MIR193BHG |
| AC018926.2 |
| AC011726.1 |
| AL358472.2 |
| TPT1-AS1 |
| AC003102.1 |
| AC021678.2 |
| AC110296.1 |
| ARRDC1-AS1 |
| LINC02580 |
| AL355377.4 |
| AL121890.2 |
| AC026369.2 |
| AC092809.4 |
| AC087286.2 |
| AC008906.1 |
| OSMR-AS1 |
| AC099791.2 |
| HID1-AS1 |
| RBM38-AS1 |
| AL590434.1 |
| ACAP2-IT1 |
| AL139147.1 |
| AL080317.2 |
| FIRRE |
| AL135791.1 |
| AC010168.2 |
| AL163051.1 |
| AC009065.5 |
| AC079414.3 |
| AC008655.2 |
| AC022613.2 |
| AC092068.1 |
| NBR2 |
| AC022506.2 |
| LINC01671 |
| AC138230.1 |
| FLJ31104 |
| AC092903.2 |
| AC148477.4 |
| EMX2OS |
| ZNF582-AS1 |
| AL590822.3 |
| AC004900.1 |
| AP003717.1 |
| LINC01137 |
| AL135790.1 |
| AC007336.2 |
| AC015853.3 |
| ERVK9-11 |
| AC010809.2 |
| SEPTIN7-DT |
| AC003093.1 |
| AC099552.3 |
| AC021148.2 |
| AC007906.1 |
| AC008750.1 |
| AL355353.1 |
| AL133342.1 |
| PPP1R14B-AS1 |
| FGF12-AS2 |
| LINC00216 |
| AL445248.1 |
| FAM66C |
| AC006042.1 |
| AL357075.3 |
| AL022328.4 |
| AC120114.1 |
| AL008718.3 |
| PGM5P4-AS1 |
| LINC00427 |
| AC023154.1 |
| AC114980.1 |
| ZNF197-AS1 |
| MIR3681HG |
| TOB1-AS1 |
| AC074194.1 |
| AC005632.3 |
| LINC01014 |
| AC026471.4 |
| AC016747.2 |
| CYTOR |
| AC008121.2 |
| AC125437.1 |
| AL031666.1 |
| AC103563.2 |
| AP000357.2 |
| DUBR |
| AC087379.2 |
| AC025171.1 |
| AC004771.4 |
| AC005954.1 |
| AC004687.1 |
| AC024060.2 |
| SLC25A21-AS1 |
| NUTM2B-AS1 |
| PCBP1-AS1 |
| AL133553.1 |
| AL356805.1 |
| AP000753.2 |
| AL359880.1 |
| AC091729.2 |
| AP000897.2 |
| FGD5-AS1 |
| AL031726.1 |
| AL390719.2 |
| AC012409.2 |
| HMGA1P4 |
| AL355075.6 |
| AC068790.6 |
| TMEM75 |
| AC018410.1 |
| MIR4458HG |
| AC074011.1 |
| AL513534.2 |
| AC020661.3 |
| AC016737.2 |
| AC064836.2 |
| LINC01004 |
| AC244034.2 |
| PHKA2-AS1 |
| AC127496.2 |
| AC007038.1 |
| AL592431.2 |
| FOXN3-AS1 |
| AC005081.1 |
| AP003307.1 |
| AC009061.2 |
| AL596094.1 |
| AC073130.2 |
| AL109615.2 |
| LIPE-AS1 |
| AL359881.1 |
| AC007601.1 |
| AC104958.2 |
| LINC00893 |
| PSMB8-AS1 |
| AC008592.4 |
| AC012313.6 |
| AC025188.1 |
| AC124066.1 |
| EPB41L4A-DT |
| LINC00174 |
| AC022034.1 |
| AC105020.2 |
| AC016026.2 |
| AC253576.2 |
| AC021483.1 |
| AL592071.1 |
| NDUFV2-AS1 |
| INE1 |
| AC099521.1 |
| AL450306.1 |
| MED8-AS1 |
| AP002433.2 |
| AP002755.1 |
| AC048344.4 |
| MBNL1-AS1 |
| AL031717.1 |
| LHFPL3-AS2 |
| AC002091.2 |
| C9orf106 |
| AC027097.1 |
| AC090912.1 |
| AC135893.1 |
| AP005329.2 |
| AL135999.1 |
| AC078955.1 |
| ZEB2-AS1 |
| ASAP1-IT2 |
| AC046143.2 |
| CADM3-AS1 |
| AL020997.2 |
| AC087392.3 |
| AC005962.1 |
| AC015818.2 |
| AP003170.3 |
| AL121999.1 |
| AC036103.1 |
| AL356010.2 |
| C10orf55 |
| LINC02100 |
| AL591848.3 |
| AL359697.1 |
| COL18A1-AS1 |
| AL391097.2 |
| AL356740.2 |
| AL008729.2 |
| AC015819.2 |
| AC004837.4 |
| AL031778.1 |
| LINC02417 |
| LINC00310 |
| AC000123.1 |
| LINC02878 |
| AC006230.1 |
| AC004846.1 |
| AC092757.2 |
| AC010998.2 |
| AC095057.4 |
| AL157834.1 |
| AC090739.1 |
| AC017100.1 |
| ZBTB20-AS1 |
| AL031768.1 |
| AC023983.1 |
| AP000866.1 |
| AL359834.1 |
| AL138921.1 |
| TH2LCRR |
| AL049838.1 |
| AL031722.1 |
| AC063965.2 |
| DLEU2 |
| AC004223.2 |
| AC109587.1 |
| MIF-AS1 |
| HOXD-AS2 |
| AL355997.1 |
| AC110015.1 |
| BX293535.1 |
| ZNF232-AS1 |
| B3GAT1-DT |
| AC138956.1 |
| AC092943.2 |
| AL121749.2 |
| GATA2-AS1 |
| CPEB2-DT |
| AC008883.1 |
| XPC-AS1 |
| AC092118.2 |
| LINC01827 |
| AL135787.1 |
| AC244033.2 |
| AL356234.3 |
| AC008946.1 |
| PCSK6-AS1 |
| TRAM2-AS1 |
| KLF3-AS1 |
| DPP9-AS1 |
| AC061992.2 |
| GAS5 |
| AC068792.1 |
| LINC02145 |
| LINC02446 |
| FALEC |
| AC009831.3 |
| LINC01355 |
| AP000254.2 |
| AP003400.1 |
| AC092119.2 |
| C3orf36 |
| MIR600HG |
| AC105020.5 |
| AC026979.1 |
| AC009159.1 |
| RUFY1-AS1 |
| AL031123.1 |
| AP000944.7 |
| AC012506.2 |
| TMED2-DT |
| AC025569.1 |
| AC117386.2 |
| AC008115.3 |
| AC073257.1 |
| LINC00476 |
| PCAT19 |
| AC008991.1 |
| AL162458.1 |
| AP003555.1 |
| AC139149.1 |
| AC098487.1 |
| AC116914.2 |
| RBM5-AS1 |
| AC011462.3 |
| LINC00265 |
| AC046134.2 |
| AL121852.1 |
| MMP2-AS1 |
| AC024267.3 |
| AL731571.1 |
| L3MBTL2-AS1 |
| AC006566.1 |
| AL136115.1 |
| AL133371.3 |
| Z99289.1 |
| AP005131.1 |
| AL161785.1 |
| AP007216.2 |
| TMEM44-AS1 |
| DIP2A-IT1 |
| AL133230.1 |
| ZNNT1 |
| AL035404.2 |
| SGMS1-AS1 |
| AC007292.2 |
| LYPLAL1-AS1 |
| ALDH1L1-AS1 |
| AF131215.5 |
| AC012459.1 |
| AC022150.2 |
| AL359921.1 |
| AC118754.1 |
| AL354977.1 |
| AL691432.2 |
| SLC2A9-AS1 |
| AL133444.1 |
| HCP5B |
| AL137779.2 |
| AC007405.3 |
| LINC02255 |
| AL139011.1 |
| AC016590.1 |
| AP003110.1 |
| AL355334.2 |
| AC010326.3 |
| AL512306.3 |
| NRXN2-AS1 |
| AC011445.1 |
| LINC02519 |
| CELF2-AS1 |
| AC004967.2 |
| AL109659.2 |
| AC138696.2 |
| AC137630.2 |
| AC040934.1 |
| AC093690.1 |
| HORMAD2-AS1 |
| AC133644.1 |
| RAMP2-AS1 |
| AC003965.1 |
| AC104088.3 |
| AC117394.2 |
| AC023051.1 |
| AC093752.1 |
| AC007255.1 |
| AC112484.5 |
| AC099811.1 |
| LINC00920 |
| AC093582.1 |
| AL034550.1 |
| RPL34-DT |
| AC084866.2 |
| AC025580.1 |
| AC145285.6 |
| AL157388.1 |
| CD27-AS1 |
| AL391261.1 |
| AC246817.2 |
| AP001178.2 |
| LINC02571 |
| Z82243.1 |
| AC004839.2 |
| AL158212.3 |
| LYST-AS1 |
| LINC02158 |
| MORF4L2-AS1 |
| LINC00582 |
| AC011603.2 |
| ATP11AUN |
| TCF4-AS2 |
| AC003086.1 |
| CAMTA1-DT |
| AC004801.6 |
| AC008543.1 |
| AL353138.1 |
| AL136320.1 |
| AL022067.1 |
| AL359704.2 |
| RNF207-AS1 |
| AL353804.1 |
| SLC25A25-AS1 |
| AC025627.1 |
| AL596244.1 |
| AC026474.1 |
| AC007881.4 |
| LINC00649 |
| AC002480.2 |
| AC097459.1 |
| BX470102.2 |
| SPINT1-AS1 |
| AC009065.2 |
| OLMALINC |
| AC078795.1 |
| AC005670.1 |
| AP000844.2 |
| AC018647.2 |
| AC002064.1 |
| AC083967.1 |
| AC024075.2 |
| AC004067.1 |
| AC243829.4 |
| AL807752.5 |
| AC022167.2 |
| ZNRF3-AS1 |
| AL121890.5 |
| AL354950.1 |
| ANKRD10-IT1 |
| SPART-AS1 |
| FOXC2-AS1 |
| LINCMD1 |
| MSC-AS1 |
| SEMA6A-AS2 |
| AC108463.3 |
| AC106795.2 |
| AC004264.1 |
| AC008764.6 |
| AC026771.1 |
| ZNF451-AS1 |
| SNHG11 |
| AC008147.2 |
| AC106820.4 |
| LINC00160 |
| ERVE-1 |
| AF117829.1 |
| AC106738.1 |
| FBXL19-AS1 |
| LINC01132 |
| AC008429.1 |
| LINC00653 |
| AL121820.3 |
| AC010525.1 |
| AC145423.1 |
| U47924.3 |
| AC135050.4 |
| SLC25A5-AS1 |
| AC020978.4 |
| AC078864.1 |
| AP002336.2 |
| AC120498.3 |
| AC020916.1 |
| AC068700.1 |
| NCKAP5-AS2 |
| GAS5-AS1 |
| AC006441.4 |
| AL117332.1 |
| Z83843.1 |
| AL358472.5 |
| AC092813.2 |
| AC007036.2 |
| AC079760.1 |
| AC016821.1 |
| AL359644.1 |
| NRIR |
| AP001099.1 |
| AC103760.1 |
| GABPB1-IT1 |
| AC092112.1 |
| AC008735.2 |
| AC023509.3 |
| AC010457.1 |
| AL021937.4 |
| BDNF-AS |
| DCST1-AS1 |
| AC090181.2 |
| AC108471.2 |
| AL050341.2 |
| ARAP1-AS2 |
| AC048341.1 |
| AC011337.1 |
| AP000892.3 |
| AC021321.1 |
| AL031668.2 |
| AL033384.2 |
| U91328.1 |
| AL357497.1 |
| ATP2B1-AS1 |
| BEAN1-AS1 |
| AL121832.2 |
| CEBPA-DT |
| AL355922.1 |
| AL358216.1 |
| AL122125.1 |
| OGFR-AS1 |
| AC007216.2 |
| BRWD1-AS1 |
| FLJ46284 |
| AC103770.1 |
| GARS1-DT |
