# Supplementary material for: Predicting prognosis, immunotherapy and distinguishing cold and hot tumors in clear cell renal cell carcinoma based on anoikis-related lncRNAs
Source: Front Immunol. 2023 Jun 9;14:1145450. doi: 10.3389/fimmu.2023.1145450 (PMC10288194; doi:10.3389/fimmu.2023.1145450)
Supplement: Supplementary file 6 [file Table_5.docx]

**Supplementary Table 5:** The lncRNAs used for modeling and the corresponding coefs.

| id | coef |
| --- | --- |
| LINC02609 | 1.159353 |
| AC007637.1 | -1.13652 |
| ELDR | 2.000735 |
| AC107021.2 | 0.261485 |
| AL022238.2 | 0.691752 |
| AC005899.7 | 0.916444 |
| LINC01522 | 1.516064 |
| MYOSLID | 0.759702 |
| AC002070.1 | -0.69125 |
| AC135178.2 | 1.381549 |
| AL590822.3 | 0.931438 |
| AL355922.1 | 0.953715 |
